# Pt–Se Hybrid Nanozymes with Potent Catalytic Activities to Scavenge ROS/RONS and Regulate Macrophage Polarization for Osteoarthritis Therapy

**DOI:** 10.34133/research.0310

**Published:** 2024-02-26

**Authors:** Hong Wei, Hongjun Huang, Haoqiang He, Yuanming Xiao, Lu Chun, Zhiqiang Jin, Hanyang Li, Li Zheng, Jinmin Zhao, Zainen Qin

**Affiliations:** ^1^ Guangxi Engineering Center in Biomedical Materials for Tissue and Organ Regeneration & Collaborative Innovation Center of Regenerative Medicine and MedicalBioResource Development and Application Co-constructed by the Province and Ministry, The First Affiliated Hospital of Guangxi Medical University, Nanning 530021, China.; ^2^ Department of Orthopaedics, Affiliated Hospital of Guilin Medical University, Guilin 541000, China.; ^3^ Life Sciences Institute, Guangxi Medical University, Nanning 530021, China.; ^4^ Department of Orthopaedics Trauma and Hand Surgery, The First Affiliated Hospital of Guangxi Medical University, Nanning 530021, China.; ^5^ School of Materials and Environment, Guangxi Minzu University, Nanning, Guangxi 53000, China.; ^6^ Guangxi Key Laboratory of Regenerative Medicine, The First Affiliated Hospital of Guangxi Medical University, Nanning 530021, China.

## Abstract

The activation of pro-inflammatory M1-type macrophages by overexpression of reactive oxygen species (ROS) and reactive nitrogen species (RONS) in synovial membranes contributes to osteoarthritis (OA) progression and cartilage matrix degradation. Here, combing Pt and Se with potent catalytic activities, we developed a hybrid Pt–Se nanozymes as ROS and RONS scavengers to exert synergistic effects for OA therapy. As a result, Pt–Se nanozymes exhibited efficient scavenging effect on ROS and RONS levels, leading to repolarization of M1-type macrophages. Furthermore, the polarization of synovial macrophages to the M2 phenotype inhibited the expression of pro-inflammatory factors and salvaged mitochondrial function in arthritic chondrocytes. In vivo results also suggest that Pt–Se nanozymes effectively suppress the early progression of OA with an Osteoarthritis Research International Association score reduction of 68.21% and 82.66% for 4 and 8 weeks, respectively. In conclusion, this study provides a promising strategy to regulate inflammatory responses by macrophage repolarization processes for OA therapeutic.

## Introduction

Osteoarthritis (OA) stands as a prevalent degenerative condition within the joint system, marked by the destruction of articular cartilage, the formation of osteophytes, and synovial inflammation [1–3]. The pathogenesis of OA is extremely complex. The current treatment focus is focusing on disease prevention, drug, or surgical treatment of early OA [4–6]. However, there are few strategies that can block or delay the development of OA. Thus, effective treatments to prevent the development and progression of OA are urgently needed.

Recent researches have demonstrated that synovial macrophages have been a key regulator in the pathological process of OA [7,8]. Macrophages can be categorized into two distinct phenotypes based on their function: the M1 subtype with pro-inflammatory effect and the M2 subtype with anti-inflammatory effect. M1 macrophages are predominantly enriched at the inflammatory site in the early stage and secrete tumor necrosis factor–α (TNF-α), interleukin-1β (IL-1β), and so on. In contrast, M2 macrophages play a crucial contribution in secreting anti-inflammatory cytokines and repairing tissue or structural reconstruction [9–11]. Particularly, activated M1 macrophages could induce the production of excess reactive oxygen species (ROS) and the release of nitric oxide (NO), which are common oxidation products and result in 30-fold higher content in comparison to M2 phenotype [12,13]. An imbalance ratio in M1/M2 macrophages was shown to be highly correlated with OA progression and severity [14,15]. Drug therapy by using antioxidants such as vitamin E [16,17], vitamin Q [18], and curcumin [19,20] demonstrated the decrease of the M1/M2 macrophage ratio by ROS or NO scavenging, leading to the deceleration of the OA progression. However, these antioxidants are broad spectrum and unspecific, with unsatisfactory stability and long-lasting therapeutic effect [21–23].

Alternatively, nanozymes with intrinsic enzyme-like activities, such as Superoxide Dismutase (SOD)-like and/or catalase (CAT)-like activities, have been widely used and exhibited superiority in ROS-related diseases in recent years [24–28]. Particularly, noble metal- or alloy nanostructure-based nanozymes, featuring the relative stable zero valence surfaces and controllable catalytic activity, attract most attention [29]. Platinum (Pt) nanoparticles (NPs), known for their exceptional catalytic properties, have been revealed to exhibit a diverse range of inherent enzyme-mimetic properties, such as SOD-, CAT-, and peroxidase (POD)-like behaviors [30,31], which can effectively scavenge free radicals into H_2_O and O_2_. It was found that Pt NPs could reduce lipopolysaccharide (LPS)-induced ROS and the expression of the pro-inflammatory cytokines, promising for OA therapy [32,33]. However, monolayer Pt NPs tends to aggregate in solution, which reduces its catalytic activities. Higher amounts of Pt may arouse acute inflammatory response and host rejection [34,35].

In recent years, hybrid composites have been shown to have higher simulation enzyme properties than mono-metal composites due to their synergistic effects, electronic structure effects, and geometric structure changes [36]. These hybrid nanomaterials have enhanced catalytic activity due to the synergistic effects between their single components. Liu et al. [37] developed functional Au@Pt core-shell NPs, which showed favorable anti-inflammatory and ROS-scavenging effects. Subramanian et al. [38] synthesized Pt-doped silver nanocomposites demonstrating dose-dependent anti-inflammatory and larvicidal (anti-dengue) activities. Sathiyaseelan et al. [39] prepared CA-mediated bimetallic AgPt NPs with unique properties to inhibit excess ROS production. However, Pt-based bimetallic hybrids are limited by unfavorable biocompatibility, poor biodegradability, and high cost since the introduction of another kind of metal [40]. Selenium (Se) is an essential trace element in human body, which is involved in regulating redox balance and plays a crucial part in governing cellular growth and apoptosis [41,42]. Se proteins in the form of selenocysteine are the most important part of the active center of selenophenase [43]. In addition, Se can be used as a doping atom commonly used in other multicomponent catalytic materials. Se modification improved stability and catalytic performance of enzymes [44–46], which has been potential therapeutics for LPS-induced inflammation [47], oxidative stress [48], and ischemic stroke [49].

**Fig. 1.**
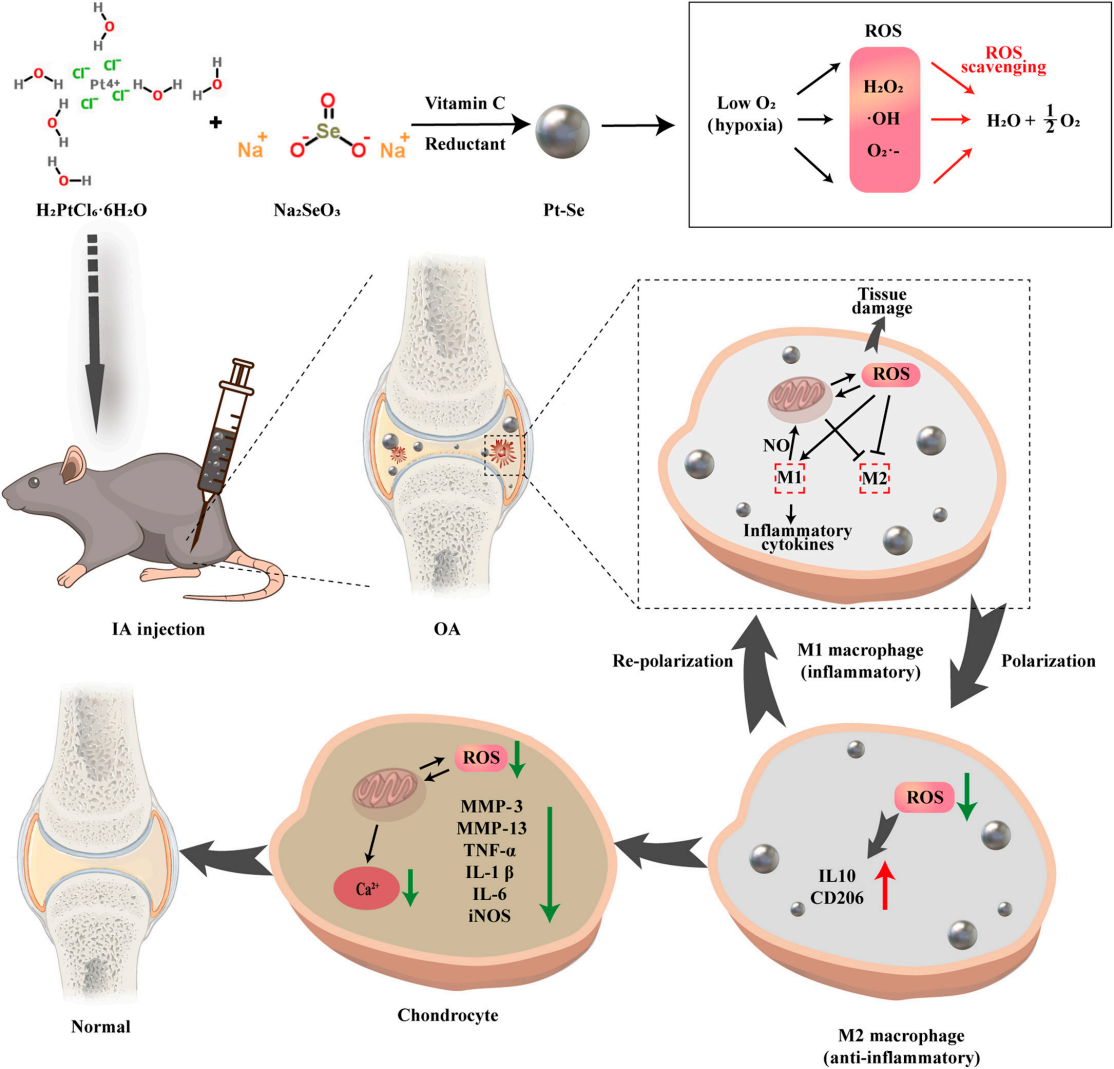
Schematic illustration of the preparation and therapeutic mechanism of Pt–Se nanozymes as SOD/CAT cascade nanozymes against OA. Pt–Se nanozymes scavenge ROS and inhibit the generation of NO to modulate the macrophage polarization from M1 to M2 in inflamed synovial joints.

In our study, we combined Pt and Se to synthesize Pt–Se composite nanozymes (Pt–Se NPs) by a simple chemical reduction method in an attempt to exert the synergistic catalytic effects on ROS and RONS (reactive nitrogen species) scavenging and macrophage repolarization in the treatment of OA (Fig. 1). This research presents a promising pathway for the treatment of OA or other chronic diseases associated with ROS by deeply exploring inherent bioactive properties of nanozymes to modify microenvironment.

## Results and Discussion

### Characterization of Se NPs, Pt NPs, and Pt–Se NPs

**Fig. 2.**
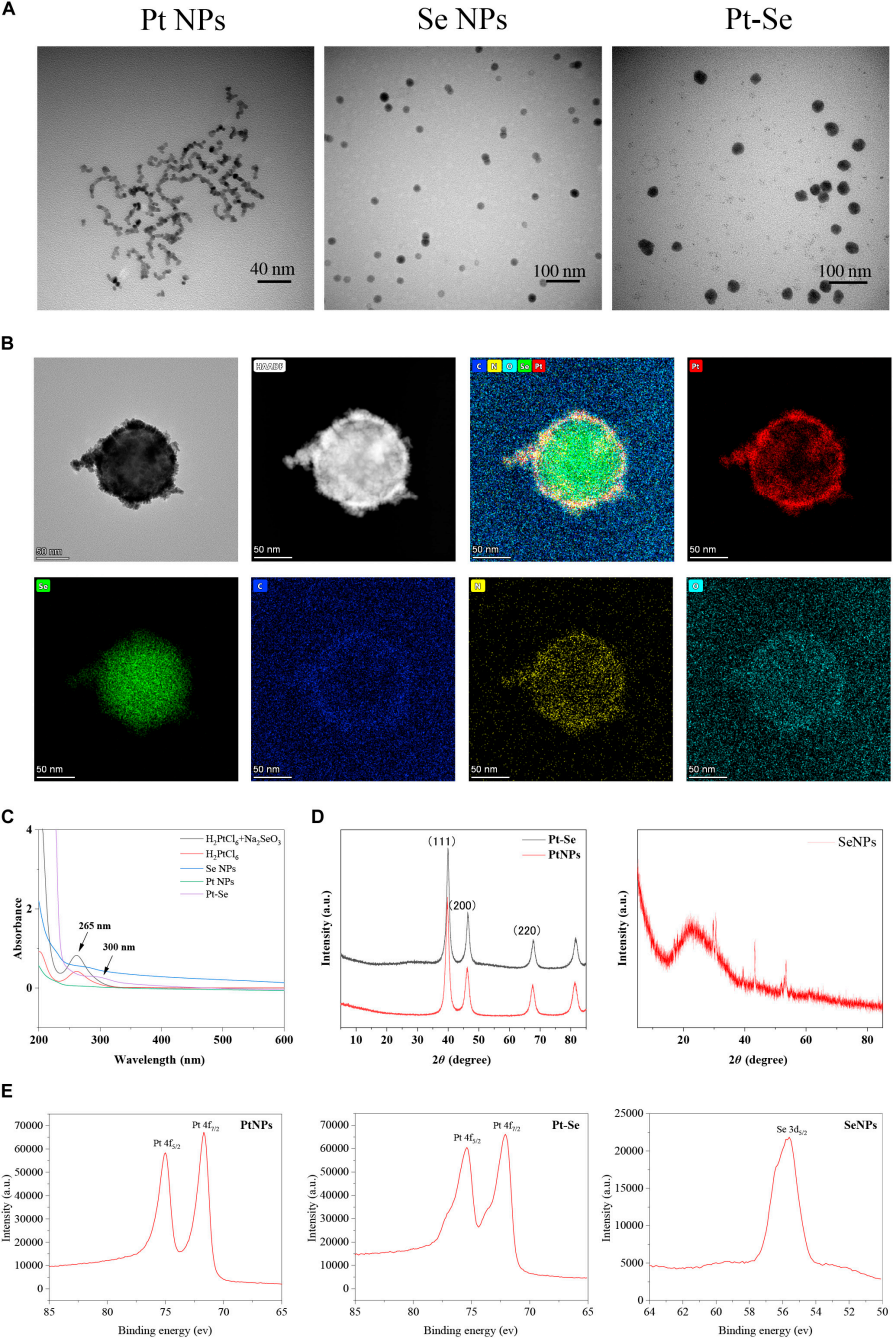
Characterization of Pt–Se NPs. (A) Representative TEM image of NPs (Pt, Se, Pt–Se). (B) TEM mapping of Pt–Se NPs: TEM and HAADF of Pt–Se; distribution diagram of Pt, Se, C, N, and O elements. (C) UV-vis spectra of Pt and Pt–Se NPs. (D) XRD results of Se NPs, Pt NPs, and Pt–Se NPs. (E) The XPS spectra were collected in both Pt and Pt–Se NPs, and Se 3d region in Pt–Se NPs.

According to previous literature, Pt, Se, and Pt–Se NPs were synthesized through a facile and optimized one-step reduction procedure [46]. The size of the Pt, Se, and Pt–Se NPs was confirmed by transmission electron microscopy (TEM) and dynamic light scattering (DLS) (Fig. 2A and Fig. S1). The results revealed that the average size of Pt NPs was 5 ± 1 nm, with poor dispersion. Se NPs exhibits spherical shape, with good dispersion and particle size of 10 nm. The size of Pt–Se hybrid structure increased to 20 ± 3 nm, a bit higher than Pt and Se. The distribution of each element on the Pt–Se hybrid NPs was confirmed by energy dispersive spectroscopy (EDS) and High-Angle Annular Dark Field Scanning Transmission Electron Microscope (HAADF). Pt–Se NPs were approximately spherical, similar to the TEM results. In addition, the Pt–Se NPs were observed to have a distribution of C, N, O, Pt, and Se elements. The weight percentage (wt%) of Pt was determined to be 7.64%, while the wt% of Se was 3.64% (Fig. 2B, Fig. S2, and Table S1). As shown in Fig. 2C, a strong ultraviolet (UV) absorption peak was observed for H_2_PtCl_6_ at 265 nm, caused by the [PtCl_6_]^2−^ ion ligand-metal charge transfer transition. The absorption spectra of the mixed solution of H_2_PtCl_6_ and Na_2_SeO_3_ were almost the same as those of the aqueous solution of H_2_PtCl_6_, indicating that H_2_PtCl_6_ did not interact with Na_2_SeO_3_ before the reaction took place. However, UV-vis spectra of Pt NPs showed that the peak value at 265 nm disappeared, indicating complete reduction of [PtCl_6_]^2−^ ions. Se NPs did not show obvious peaks. Compared with Pt NPs, the UV absorption peak of Pt–Se NPs at 265 nm also vanished, with a new absorption peak at 300 nm, which was caused by the vibration of the Se NPs. In Fig. 2D, the XRD patterns of Pt, Se, and Pt–Se NPs were observed. The diffraction peaks exhibited by Pt NPs matched the (111), (200), and (220) lattice planes, in agreement with previous measurements reported in JCPDS 80-1268 [50]. There was a small right shift in the (111), (200), and (220) lattice planes of the Pt–Se nanozymes spectrum, which may be caused by the introduction of Se. Similar to pure Pt NPs, Pt–Se nanozymes containing doped Se may possess crystal structures. There is no typical diffraction peak of crystal Se in the XRD pattern of Pt–Se, indicating that the Se element in Pt–Se may exist in the amorphous form [51]. To confirm the composition of Pt–Se nanozymes, XPS spectra were collected from the Pt 4f region and Se 3d region. The characteristic binding energies of Pt 4f_7/2_ were measured to be 71.5 eV, while the binding energy for Pt 4f_5/2_ was found to be 75.0 eV (Fig. 2E), indicating the reduction of Pt (0). An XPS spectrum of the Pt 4f region of Pt–Se nanozymes indicates Pt 4f_7/2_ peaks at about 72.1 eV and Pt 4f_5/2_ peaks at about 75.4 eV, indicating that it contains element Pt (0). In Pt–Se NPs, the 3d orbit binding energy of Se in the 3d region measured by XPS was found to be about 55.6 eV. Thus, the Pt–Se nanozymes contain Pt in its reduced state (Pt^0) and Se in its reduced state (Se^0). All the above results indicate structural composition of Pt–Se, demonstrating the successful synthesis.

### Free radical scavenging ability of Pt–Se NPs

**Fig. 3.**
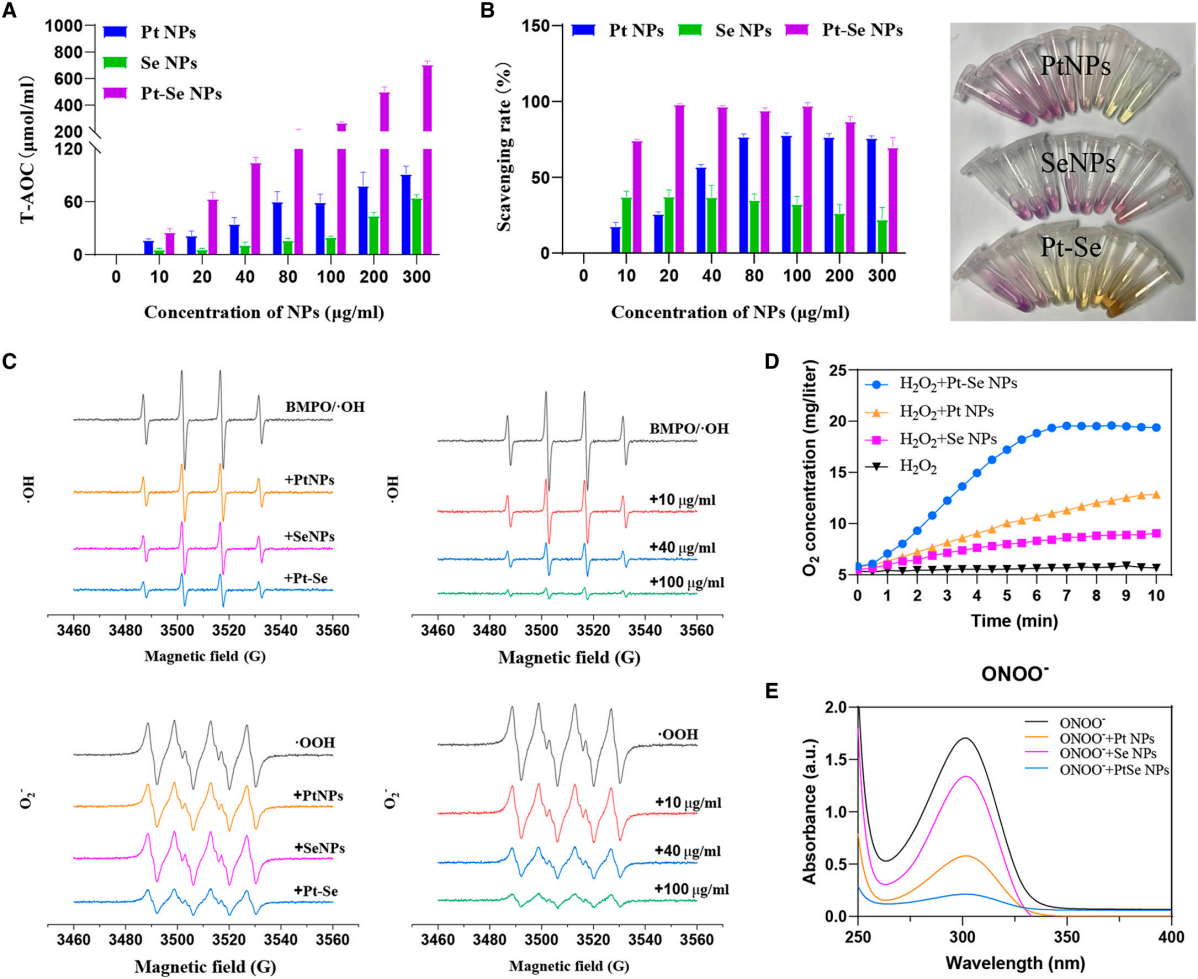
ROS/RONS-scavenging activities of Pt–Se NPs. (A) Total anti-oxidation capability test of Pt, Se, and Pt–Se NPs. (B) DPPH radical-scavenging capacity of Pt, Se, and Pt–Se NPs, and color change of DPPH reagent after treatment with NPs. (C) ·OH-scavenging ability of Pt, Se, and Pt–Se at the same concentration, and ·OH-scavenging ability of Pt–Se at different concentrations; O_2_^·−^ scavenging ability of Pt, Se, and Pt–Se NPs at the same concentration, and O_2_^·−^ scavenging ability of Pt–Se at different concentrations. (D) Determination of the Pt–Se NPs with catalase-like activity. (E) ONOO^−^-scavenging activity of the Pt–Se NPs.

In inflammatory reactions, several types of ROS/RONS, which include hydrogen peroxide (H_2_O_2_), hydroxyl radical (·OH), superoxide anion (O_2_^·−^), ·NO and ONOO^−^, and other free radicals, can cause tissue damage [52–54]. Additionally, excessive oxidative stress is also an important pathogenesis factor for OA [55]. Therefore, eliminating multiple ROS/RONS and reducing oxidative stress responses are necessary to protect tissues from inflammatory damage. To assess the total antioxidant capacity (T-AOC) of Pt–Se, a T-AOC assay kit was utilized. As shown in Fig. 3A, the T-AOCs of Pt, Se, and Pt–Se NPs increased with the concentrations. At concentration of 100 μg/ml, Pt–Se showed a higher level of T-AOC (300 μmol/ml) than Pt NPs and Se NPs, demonstrating a better T-AOC. We further assessed their abilities to scavenge 2,2-diphenyl-1-pyridinohydrazide (DPPH) and other kinds of ROS (H_2_O_2_, ·OH, and O_2_^·−^). It was found that the scavenging ability of DPPH depends on the concentration of Pt–Se NPs. The maximum DPPH-scavenging capacity of 20 μg/ml Pt–Se exceeded 90% (Fig. 3B), much higher than Pt NPs (~30%) and Se NPs (~40%), indicating that Pt–Se NPs were more efficient in eliminating DPPH than Pt or Se NPs. The electron paramagnetic resonance (EPR) signal obtained from the spin adducts 5-tert-butyl carbyl-5-methyl-1-pyrroline *N*-oxide (BMPO)/·OH and DEPMPO/·OOH exhibited a notable decrease as the concentration of nanozyme increases, suggesting an enhanced radical quenching capacity for hydroxyl radical (·OH) and superoxide anion (O_2_^·−^) by the Pt–Se nanozymes. At the same concentration of 40 μg/ml, a noticeable decrease in the EPR signal was observed in the Pt–Se nanozymes compared to the individual Pt and Se NPs, illustrating a superior scavenging effect on ·OH and O_2_^·−^ (Fig. 3C). Furthermore, as depicted in Fig. 3D, the O_2_ production results indicated a catalase-like activity of the Pt–Se NPs. Remarkably, the catalytic efficiency of the Pt–Se NPs surpassed that of both Pt NPs and Se NPs. Additionally, it was revealed that the Pt–Se NPs exhibited a remarkable scavenging activity for ONOO^−^ in vitro (Fig. 3E). These findings underscore the effective enhancement of antioxidant and ROS/RONS-scavenging capabilities in Pt–Se hybridization compared to Pt or Se NPs.

### The toxicity assessment of Pt–Se NPs

**Fig. 4.**
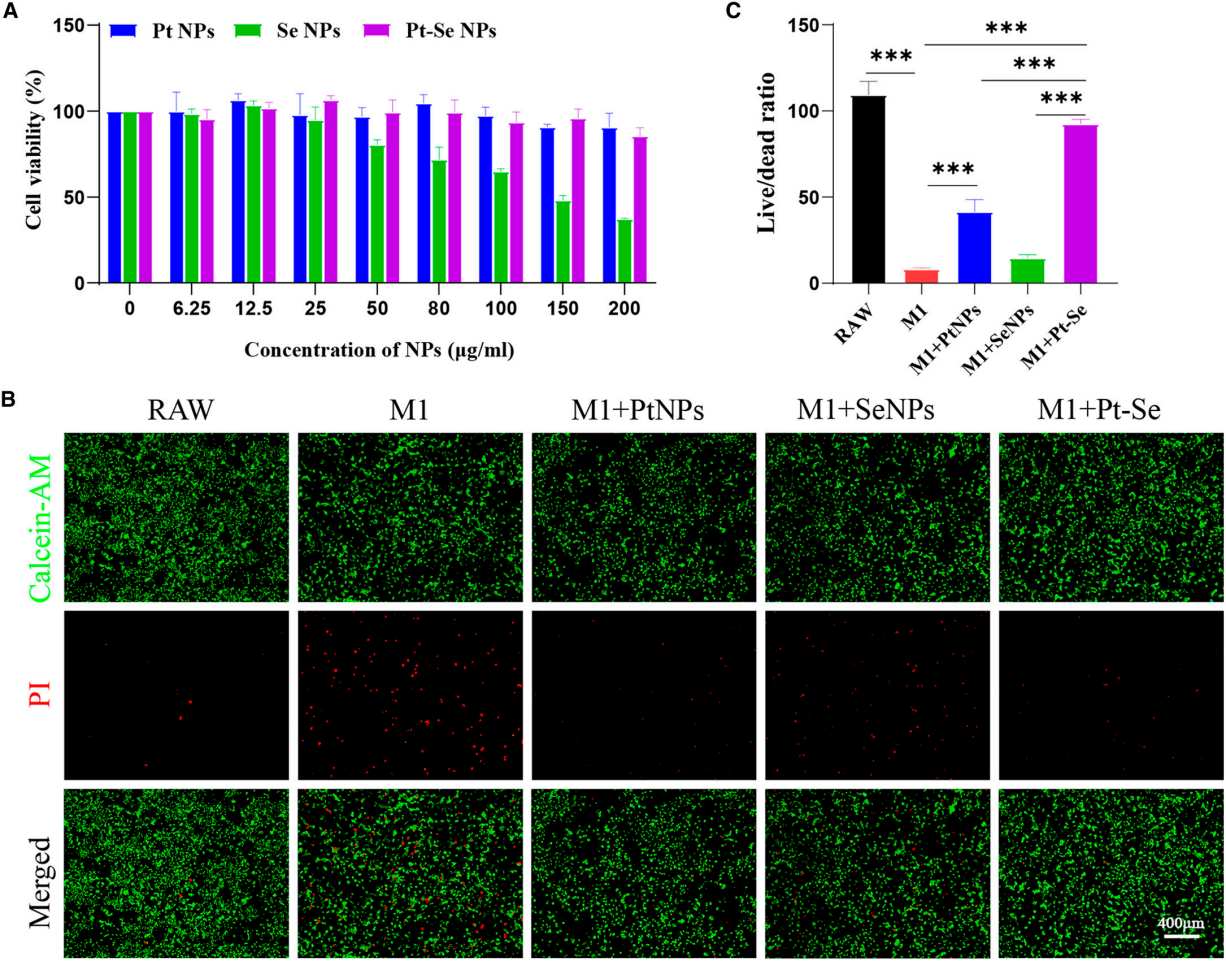
Toxic effects of nanoparticles on macrophages. (A) Cytotoxicity of Pt, Se, and Pt–Se NPs to RAW264.7 as evaluated by CCK-8 assay. (B) Calcein-AM/PI staining was performed, and the live/dead cell ratio was quantified (C). Scale bar, 400 μm. *n* = 3. The symbol “*” indicates a comparison between groups. ^***^P < 0.001.

The biocompatibility of Se, Pt, and Pt–Se NPs on RAW264.7 was evaluated by CCK-8 assay. As shown in Fig. 4A at the concentration of 100 μg/ml, the cell viability of Se decreased to 80%, while both Pt and Pt–Se NPs consistently retained a cell viability above 95%. Thus, 100 μg/ml of Pt–Se NPs was chosen as the final concentration for subsequent experiments. Further, we investigated the effect of Pt–Se NPs on survival of RAW264.7 cells by using calcein-AM/propidium iodide (PI) staining. As illustrated in Fig. 4B, LPS stimulation resulted in an increase in dead cells (red) and a decrease in live cells (green) in comparison to normal RAW. However, a large number of live cells were observed after Pt, Se, and Pt–Se NP treatment, especially in the Pt–Se NP group. Thus, Pt–Se NPs have no obvious toxicity and could rescue the LPS-reduced cell viability in vitro, making it promising for further biomedical applications. In addition, we found that Pt–Se could be well taken up by cells through cellular uptake assay (Fig. S4).

### Intracellular ROS and NO levels regulated by Pt–Se NPs

**Fig. 5.**
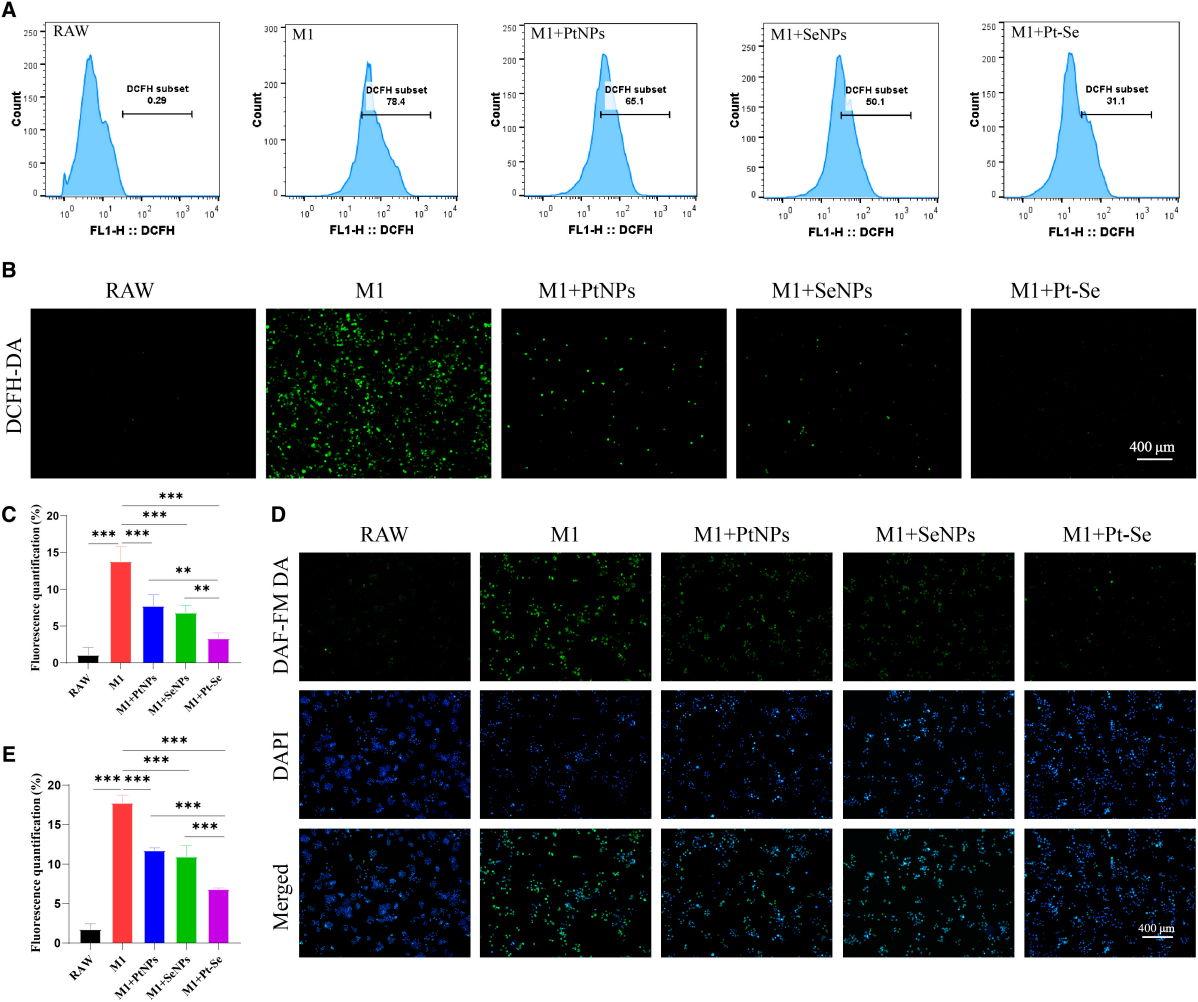
Effects of NPs on intracellular ROS and NO. (A) Flow cytometry analysis and fluorescence images (B) of intracellular ROS levels in RAW264.7 cells by a DCFH-DA probe after treatment with NPs. (C) Relative quantitation of ROS-scavenging ability by fluorescent images. (D) The DAF-FM DA probe was utilized to detect the levels of NO in macrophages, and the relative quantitative of NO was measured (E). Original magnification, 100×. Scale bar, 400 μm. *n* = 3. ^**^P < 0.01, ^***^P < 0.001.

The level of ROS and NO mediated in oxidative stress is generally increased in inflamed joints, resulting in chondrocyte apoptosis and cartilage extracellular matrix (ECM) degeneration [56]. Thus, scavenging excessive ROS and NO contributes to relief of OA progression. We studied the ROS- and NO-scavenging ability of Pt–Se NPs in vitro using LPS-activated RAW264.7 macrophages, which could create an inflammatory microenvironment. The fluorescence of DCFH-DA (2′,7′-dichlorodihydrofluorescein diacetate) represented by the total intracellular ROS level analyzed by FACS increased to 78.11% after LPS stimulation (Fig. 5A). However, the ROS levels were remarkably decreased in Pt, Se, and Pt–Se NPs, compared with the RAW group. In particular, the Pt–Se NPs showed the highest scavenging of ROS, with a 47.3% reduction. These results were consistent with those obtained by fluorescent microscopy (Fig. 5B and C). The similar results were also confirmed by NO-scavenging assay with a DAF-FMDA kit (NO fluorescent probe). As shown in Fig. 5D and E, NO levels in RAW264.7 macrophages were increased compared to those without LPS stimulation. Pt and Se NPs markedly reduced the NO production level in LPS-activated RAW264.7 macrophages. In contrast, Pt–Se NPs remarkably reduced the level of NO to 6.45%. All these data indicated that Pt–Se NPs could reduce intracellular oxidative stress levels by clearing intracellular ROS and inhibiting NO production.

### Effect of Pt–Se NPs on repolarization of macrophages

**Fig. 6.**
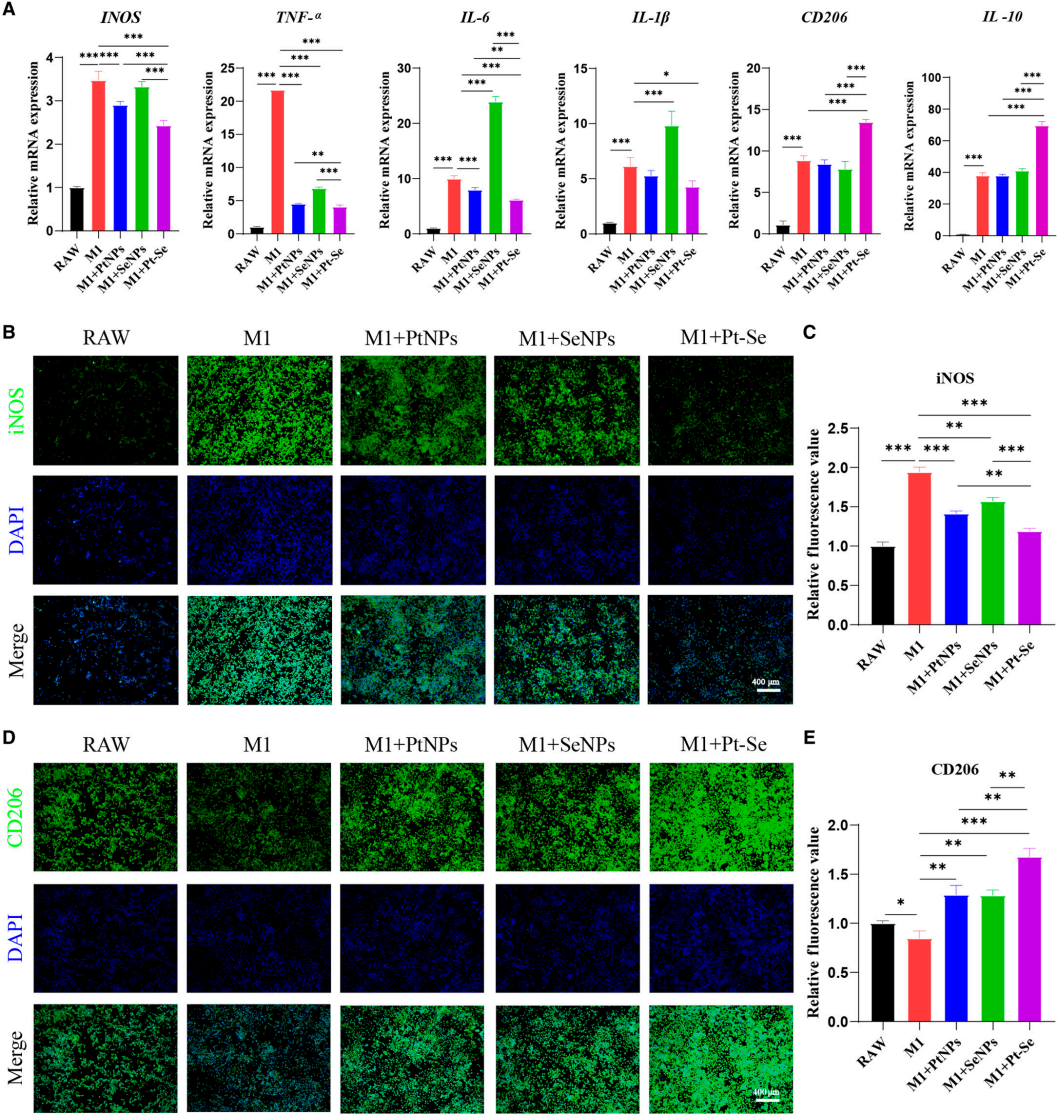
In vitro effects of NPs on macrophage repolarization. (A) The relative gene expression levels of iNOS, TNF-α, IL-1β, IL-6, CD206, and IL-10 in macrophages were detected by qRT-PCR. The secretion of macrophage-related proteins iNOS (B and C) and CD206 (D and E) was detected by immunofluorescence staining and the corresponding fluorescence quantity. Original magnification, 100×. Scale bar, 400 μm. *n* = 3. ^*^P < 0.05, ^**^P < 0.01, ^***^P < 0.001.

Macrophages, particularly the M1 phenotype, as important inflammatory cells in the synovial membrane and synovial fluid, play a crucial role in the progression of OA [57]. To verify the anti-inflammatory effects of Pt–Se NPs on macrophage repolarization, the expression of M1 markers such as inducible nitric oxide synthase (iNOS), IL-6, TNF-α, and IL-1β, along with M2 markers CD206 and anti-inflammatory cytokine IL-10, was investigated by quantitative reverse transcription polymerase chain reaction (qRT-PCR) (Fig. 6A). Those inflammatory cytokines secreted by M1 macrophages were significantly increased after LPS treatment. Following treatment with Pt–Se NPs, the expression of these pro-inflammatory factors decreased significantly, while the mRNA levels of M2 marker CD206 and anti-inflammatory cytokine IL-10 were effectively enhanced in LPS-activated macrophages. Furthermore, the repolarization of macrophages by Pt–Se NPs was also verified by immunofluorescence imaging. Figure 6B and C demonstrates a significant increase in the expression level of iNOS in RAW264.7 macrophages activated by LPS (increased by about 48.5 ± 1.2% compared to the blank group), indicating the polarization toward the M1 phenotype. The iNOS levels were decreased by 28.3 ± 8.8% and CD206 levels were increased to 39.3 ± 8.6% remarkably after NP treatment, indicating successful repolarization from M1 to M2 phenotype. Particularly, Pt–Se NPs exhibited the most marked inhibition of iNOS expression and the highest promotion of CD206 expression, consistent with the results of qRT-PCR analysis, promising for protection of cartilage matrix.

### Pt–Se NPs protecting chondrocytes in inflammatory condition

**Fig. 7.**
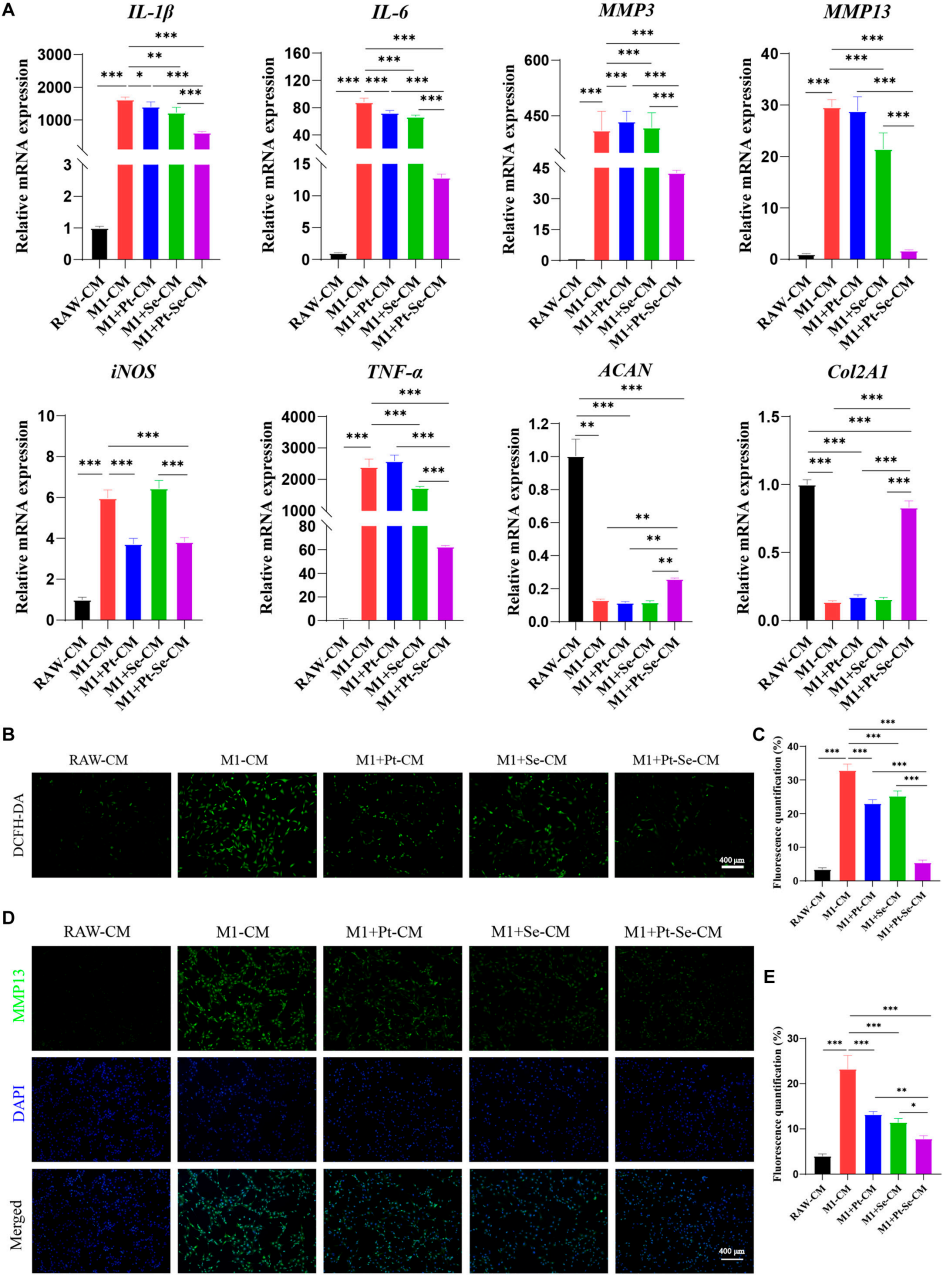
Effects of NPs on chondrocytes. (A) qRT-PCR assay of IL-1β, IL-6, MMP-3, MMP-13, iNOS, TNF-α, ACAN, and Col2A1 in chondrocytes stimulated with the macrophage CM. (B) A DCFH-DA probe was utilized to detect the levels of ROS in chondrocytes exposed to the macrophage CM. (C) Quantitative analysis of intracellular ROS. (D) The secretion of MMP-13 in chondrocytes exposed to macrophage CM was measured by immunofluorescence staining and quantitative analysis of fluorescence intensity (E). Original magnification, 100×. Scale bar, 400 μm. *n* = 3. ^*^P < 0.05, ^**^P < 0.01, ^***^P < 0.001.

The activation of M1 macrophages leads to the release of substantial quantities of chemokines and pro-inflammatory factors, such as TNF-α, IL-6, and IL-1β [15], resulting in the infiltration of inflammatory cells and a large amount of pro-inflammatory factor accumulation in OA joint, thus degrading ECM and aggravating cartilage damage [15]. Meanwhile, activated M1 macrophages also produce a large number of ROS, resulting in the extracellular toxicity and the up-regulation of pro-inflammatory cytokine production. Cell survival was approximately 80% when Pt–Se NP concentration was 100 μg/ml, indicating that NPs did not inhibit chondrocyte viability at the concentration used to treat macrophages (Fig. S3A). Furthermore, no significant cellular degeneration or inflammation was observed after 24 h of incubation with NPs and chondrocytes (Fig. S3B). These results suggest that Pt, Se, and Pt–Se NPs did not directly affect chondrocytes. It has been reported that imbalance between catabolism and anabolism contributes to the progression of OA [15,58]. We further investigated the expression of key genes related to inflammation, including matrix metalloproteinase-3 (MMP-3), MMP-13, IL-1β, IL-6, iNOS, and TNF-α, as well as cartilage-specific markers such as Col2α1 and Acan (Fig. 7A). Results showed the IL-1β, IL-6, MMP-3, MMP-13, iNOS, and TNF-α expression levels were elevated after pretreatment with M1-CM, accompanied by a decrease in the cartilage-specific markers (Col2α1 and Acan), suggesting that the M1 phenotype could promote chondrocytes in a degenerative state. The expression levels of those inflammatory markers were significantly inhibited by M1 + Pt NP-CM, M1 + Se NP-CM, and M1 + Pt–Se NP-CM. Particularly, M1 + Pt–Se NP-CM showed the highest decrease in these inflammation-related cytokine expression level in all the groups (*P* < 0.05). The gene expression of ACAN and Col2A1, specific for articular cartilage, was promoted especially in M1 + Pt–Se NP-CM (*P* < 0.05). This indicates that Pt–Se NPs exhibited a robust ability to attenuate the progression of OA. To assess the ROS content in chondrocytes following exposure to different conditioned media (CM), we utilized DCFH-DA staining. As depicted in Fig. 7B and C, chondrocytes cultured in RAW-CM exhibited a low level of ROS production. Conversely, when exposed to M1-CM, chondrocytes displayed a significant increase in ROS generation. Chondrocytes cultured in M1 + Pt–Se NP-CM showed significantly decreased ROS signals compared with M1 + Pt NP-CM and M1 + Se NP-CM, indicating the strong ROS-scavenging capacity of Pt–Se NPs. Immunofluorescence analyses also showed that M1 + Pt–Se NP-CM significantly down-regulated CM-induced MMP-13 release (Fig. 7D and E). These results suggested that Pt–Se exhibits anti-inflammatory and chondro-protective effects by reversing damage caused by M1 macrophages and supporting chondrocytes growth, creating a healthier microenvironment for tissue healing.

### Pt–Se NPs protecting mitochondrial function recovery in inflammatory chondrocytes

**Fig. 8.**
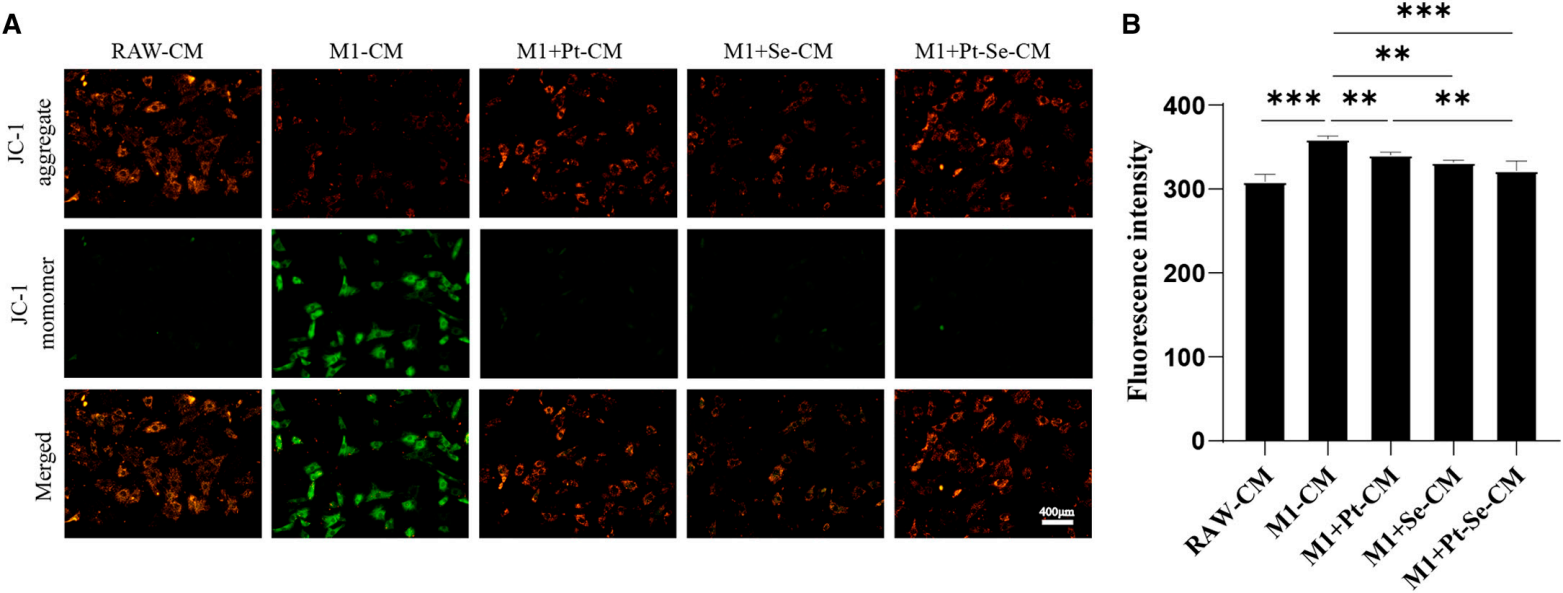
Intracellular mitochondrial membrane potential and calcium ion levels in chondrocytes. (A) The JC-1 probe was employed to detect the mitochondrial membrane potential in chondrocytes. (B) A Fluo-3 AM probe was used to detect the calcium ion levels in chondrocytes treated with macrophage CM. Original magnification, 100×. Scale bar, 400 μm. *n* = 3. ^**^P < 0.01, ^***^P < 0.001.

During OA progression, the increase of the ROS level could induce the imbalance of Ca^2+^ homeostasis in chondrocyte mitochondria, resulting in a large amount of extracellular Ca^2+^ inflow so as to “Ca^2+^ overload,” which disrupts the normal function of mitochondrial, cell, or tissue [59]. We tested the mitochondrial membrane potential in chondrocytes with a JC-1 Assay kit. According to the results (Fig. 8A), the M1 + Pt–Se-CM group had weaker green fluorescence intensity and more intense red fluorescence than the other groups, indicating that Pt–Se NPs could better improve the mitochondrial membrane potential than Pt and Se NPs. In addition, Fluo-3 AM probe was used to detect the level of Ca^2+^ in chondrocytes (Fig. 8B), and the results showed that NPs could reduce the abnormal accumulation of Ca^2+^ in the cells, especially in the M1 + Pt–Se-CM group. All these results indicated that Pt–Se NPs may save mitochondrial function and reduce chondrocyte injury.

### Pt–Se NPs attenuate the progression of OA in vivo

Based on anterior cruciate ligament transection (ACLT)–induced OA joint model, we further investigated the potency of Pt–Se NPs for OA treatment in vivo. First, the in vivo longevity of Pt–Se NPs was evaluated through an in vivo imaging system on days 0, 1, 3, 5, 7, and 14. As depicted in Fig. S5, the fluorescence intensity in the Pt–Se/DSPE-Cy5.5 group surpassed that in the free Cy5.5 group at various time intervals. Nevertheless, free Cy5.5 exhibited a decline in fluorescence intensity over time, and by day 14, no discernible fluorescence was observed, indicating a considerable duration of retention within the articular cavity.

**Fig. 9.**
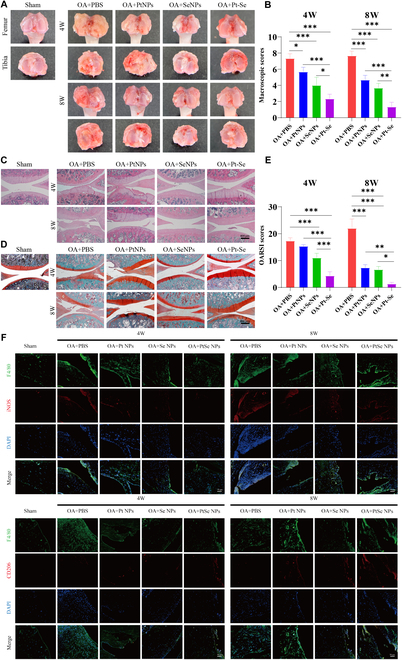
Effects of NPs on OA in vivo. Macroscopic observation (A) and corresponding macroscopic scores (B) of OA articular cartilage after 4 and 8 weeks of treatment with the intra-articular injection of NPs. H&E staining (C), S&F (D), and corresponding histological scores (E) of articular cartilages after treatment with NPs. (F) The polarization of macrophages in joint synovial membrane was observed by immunofluorescence staining. Macrophages were labeled with F4/80 (green), and M1-type macrophage-related markers were detected by iNOS (red), while M2-type macrophage-related markers were detected by CD206 (red). Original magnification, 100×. Scale bar, 400 μm. *n* = 3, ^*^P < 0.05, ^**^P < 0.01, ^***^P < 0.001.

At 30 days after surgery, all groups of rats were administrated and repeated twice a week until 8 weeks. We assessed the therapeutic effects of NPs on OA by the macroscopic observation and the score of the cartilages from the tibial plateau and distal femur. There were rough and erosive surface of the articular cartilage, osteophytes, and synovium hyperplasia, the characteristics of OA, which worsened with time in OA groups (Fig. 9A). The recovery and score of cartilage lessons was observed in the order of Pt > Se > Pt–Se (Fig. 9B). Particularly, Pt–Se showed the most obvious repair effect with the smooth and integrated surface, with small cracks and osteophytes. Pt–Se displayed the most markedly therapeutic efficiency on OA compared to all the groups, with the macroscopic scores decreasing to 68.21% and 82.66% at 4 and 8 weeks, respectively.

The histological examination was also used to analyze the therapeutic efficiency of NPs on OA. As shown in Fig. 9C, hematoxylin and eosin (H&E) staining results showed clear inflammatory hyperplasia, unclear cartilage boundaries, fibrillation, loss of proteoglycans, and deformation of cartilage, which deteriorated over time in the OA group, in contrast to smooth cartilage surface in sham rats. In contrast, treatment with Pt, Se, or Pt–Se NPs prominently ameliorated the morphology, as demonstrated by smoothness of cartilage surface, tidemark integrity, and columnar structure of chondrocytes. Particularly, Pt–Se NP administration resulted in the retention of a well-preserved cartilage surface close to an intact cartilage structure. Furthermore, Safranin O-fast green staining (S&F) was conducted to evaluate the content of glycosaminoglycan (GAG) in the cartilage affected by OA (Fig. 9D). The S&F clearly demonstrated that the sham group exhibited a high density of GAG, with positive staining. In contrast, most GAG was lost in OA rats as a result of extensive cartilage deterioration, with GAG-negative signal. However, Pt, Se, and Pt–Se treatment effectively mitigated the severity of cartilage damage. Among these treatments, Pt–Se intervention demonstrated the highest preservation of GAG content, which was even comparable to that of the sham group. The histological score, as depicted in Fig. 9E, confirmed the superior therapeutic effects of Pt–Se NPs. Among the four ACLT groups, the Pt–Se group exhibited the lowest histological score. In addition, the polarization of macrophages in synovial tissues was observed by immunofluorescence staining of synovial tissues. The results revealed a high expression of the M1-type macrophage marker iNOS in the OA group. On the other hand, the administration of Pt–Se treatment led to an increased expression of the M2-type macrophage marker CD206 in synovial tissue, while the expression of iNOS, the marker for M1-type macrophages, significantly decreased compared to the other two groups (Fig. 9F). These results suggested that the administration of Pt–Se NPs could facilitate the polarization of macrophages in the synovial membrane toward the M2 type and prolong their presence within the joint cavity, consequently alleviating OA-associated joint injuries.

## Conclusions

We successfully prepared Pt–Se hybrid nano-enzyme by taking advantage of the exceptional catalytic properties of Pt and Se to exert synergistic effects. The findings of the study revealed that Pt–Se had good antioxidant and anti-inflammatory activities and low cytotoxicity. The hybridization of Se not only reduces the usage of Pt but also enhances the free radical scavenging ability. In addition, it can effectively promote the transition of M1 macrophages into anti-inflammatory M2-phenotype by scavenging excess ROS and RONS, achieving a more suitable microenvironment for articular chondrocytes. At the same time, it can reduce mitochondrial damage and protect chondrocytes. The in vivo study also demonstrated that Pt–Se NPs substantially contributed to cartilage repair in OA. In general, this study provides a novel platform for the treatment of inflammation disease associated with high oxidative stress.

### Materials and Methods

### Materials

Hexachloroplatinic acid (H_2_PtCl_6_^.^6H_2_O), NaBH_4_, ascorbic acid, Na_2_SeO_3_, and polyvinylpyrrolidone (PVP) were provided by Aladdin (Shanghai, China). Glutathione (GSH), DCFH-DA probe, Total RNA Extraction Kit, penicillin-streptomycin solution, EDTA decalcified fluid, H&E Staining Kit, and S&F were acquired from Solarbio Co. Ltd. (Beijing, China). LPS and Cell Counting Kit-8 (CCK-8) were obtained from Biosharp Co. Ltd. (Beijing, China). Calcein-AM/PI and DPPH were from Sigma-Aldrich Co. Ltd. (USA) and TCI (Shanghai, China), respectively. Furthermore, Mitochondrial Membrane Potential Assay Kit with JC-1, a 3-amino,4-aminomethyl-2′,7′-diflfluorescein diacetate (DAF-FM DA) probe, a Nitric Oxide Synthase Assay Kit, and a T-AOC assay kit, Fluo-3 AM, were purchased from Beyotime Co. Ltd. (Shanghai, China). Fetal bovine serum (FBS), Dulbecco’s modified Eagle’s medium (DMEM), iNOS and CD206 antibodies, qPCR Detection Kit, and First Strand cDNA Synthesis Kit were acquired from Gibco Life Technologies Inc. (Grand Island, NY, USA). Arg-1, CD206, IL-10, IL-1β, iNOS, TNF-α, IL-6, and GAPDH (glyceraldehyde-3-phosphate dehydrogenase) primers were designed by Genecreate Co. Ltd. (Wuhan, China).

### Cells

Murine RAW264.7 macrophage cells were purchased from Procell (Wuhan, China), and primary chondrocytes were isolated from the articular cartilage of 3- to 5-day-old Sprague-Dawley rats, which were provided by the Laboratory Animal Center of Guangxi Medical University. Those cells were incubated at 37 °C with fresh DMEM containing 10% FBS, 1% penicillin (50 U/ml), and streptomycin (50 U/ml) in a 5% CO_2_ atmosphere.

### Synthesis of the Se NPs, Pt NPs, and Pt–Se NPs

In order to produce Se NPs, 400 mg of bovine serum albumin (BSA) was mixed completely with 20 ml of 10 mM GSH. After BSA was completely dissolved, 5 ml of 10 mM Na_2_SeO_3_ aqueous solution was added and fully stirred. A pH adjustment of 7.6 was achieved by adding 1 M NaOH. As a result of the addition of NaOH, the solution color changes rapidly to orange-red, indicating the formation of Se NPs. Then, dialysis of the prepared Se NP colloid solution was performed using a dialysis bag with a molecular weight cutoff of 3,500. The dialysis device was dialyzed in distilled water for 48 h, and the dialysis water was changed every 24 h during the process. In order to obtain red Se NP precipitates, the colloidal solution containing Se NPs was centrifuged at high speed at 10,000 rpm after dialysis. The Se NP precipitates were re-dispersed in tertiary water and stored at 4 °C.

Pt NPs were synthesized as follows: A mixture of 5 ml of 5 mM H_2_PtCl_6_ and 50 mM NaBH_4_ was added at room temperature under vigorous stirring. The reaction solution was reconstituted with doubled-distilled water to a volume of 50 ml. The reaction solution continued for 30 min at room temperature, and the mixed solution turned brown, indicating that Pt NPs had formed. Ascorbic acid is dissolved in 35 ml of doubled-distilled water, followed by 5 ml of H_2_PtCl_6_, 5 ml of Na_2_SeO_3_, and a small amount of PVP to make Pt–Se nanostructures. As a result of stirring the mixture at room temperature for 2 h, the reaction system turned brown and black, which indicated the formation of nanostructures of Pt–Se. Using ethanol and acetone, excess PVP was removed from the colloidal solution containing Pt and Pt–Se NPs. In order to prepare a stock solution of Pt and Pt–Se NPs from the precipitate, we resuspended it in double distilled water.

### Characterizations of Se NPs, Pt NPs, and Pt–Se NPs

A variety of Se NPs, Pt NPs, and Pt–Se NPs were characterized as follows: transmission electron microscopy (TEM, Bruker, Germany), DLS (Malvern, UK), high-resolution TEM (HRTEM, FEI Talos F200X, Thermo Fisher, USA), x-ray powder diffraction (XRD, Bruker, Germany), x-ray photoelectron spectroscopy (XPS; Thermo Fisher Scientific, USA), and UV-vis absorption (Shimadzu, Japan).

### Free radical scavenging activity assay

#### T-AOC test

The T-AOC of Se NPs, Pt NPs, and Pt–Se NPs was assessed by a T-AOC Assay Kit. Briefly, Pt, Se, and Pt–Se NPs at series concentrations with 0, 10, 20, 40, 80, 100, 200, and 300 μg/ml were prepared. The test of T-AOC was conducted according to the directions of the T-AOC kit.

#### DPPH-scavenging capacity

The DPPH-scavenging capacity of Pt–Se was tested by DPPH method. In a nutshell, 400 μl of DPPH solution was added into 200 μl of Pt, Se, and Pt–Se NPs with different concentrations (0, 10, 20, 40, 80, 100, 200, and 300 μg/ml), mixed at room temperature for 10 min, and then centrifuged at 10,000 rpm for 5 min. An absorbance measurement at 540 nm was performed on the supernatant.

#### Scavenging activity against hydroxyl radicals

The hydroxyl radical (·OH)-scavenging capacity of Se, Pt, and Pt–Se NPs was assessed using EPR (Talos F200X, Thermo Fisher, USA). Hydroxyl radicals were produced by irradiation of 5 mM H_2_O_2_ in 10 mM buffer for 5 min. BMPO (50 mM) was used to capture hydroxyl radicals and generate spin adduct (BMPO/·OH). EPR spectra of Pt, Se, and Pt–Se NPs (40 μg/ml) at the same concentration were recorded at 0.6 mW microwave power and 1-G modulation amplitude. In addition, the EPR spectra of Pt–Se NPs at different concentrations (40 and 100 μg/ml) were detected.

#### Determination of H_2_O_2_-scavenging ability

The H_2_O_2_-scavenging activity of the material was evaluated by quantifying the oxygen level generated using the Dissolved Oxygen Meter (JPSJ-605F, INESA, China). Equal concentrations of Pt, Se, and Pt–Se NPs were individually combined with 10 mM H_2_O_2_. The resulting oxygen was automatically detected by the dissolved oxygen meter at 10-s intervals for a total duration of 20 min.

#### Determination of ONOO^−^ scavenging activity

A mixture of NaNO_2_ (10 ml, 50 mM) and H_2_O_2_ (10 ml, 50 mM) was vigorously agitated in an ice water bath. Afterward, HCl (5 ml, 1 M) and NaOH (5 ml, 1.5 mM) were introduced into the mixture, resulting in the formation of a yellow solution containing ONOO^−^. Following this, the reaction substrate solution was diluted 10 times to obtain the final reaction solution. Subsequently, Pt, Se, and Pt–Se NPs of equal concentration were added to initiate the reaction. The absorbance of ONOO^−^ at 302 nm was measured using UV-vis spectroscopy, and the changes in the absorption spectrum over time were recorded.

#### SOD-mimetic catalytic activity

The superoxide (O_2_^·−^) scavenging capacity of Se, Pt, and Pt–Se NPs was investigated using EPR. Potassium superoxide (KO_2_) and 5-(diethoxyphosphoryl)-5-methyl-1-pyrroline-*n*-oxide (DEPMPO) were used as the source and spin well of O_2_^·−^, respectively. A solution of dimethyl sulfoxide (DMSO) containing 2.5 mM KO_2_, 25 mM BMPO, and 3.5 mM 18-crown-6 was finally obtained. EPR spectra of Pt, Se, and Pt–Se NPs (40 μg/ml) at the same concentration were recorded. In addition, the EPR spectra of Pt–Se NPs at different concentrations (40 and 100 μg/ml) were detected.

### In vitro cell viability test

For 24 h, we activated RAW264.7 and cartilage cells with LPS (10 μg/ml) in 96-well plates. After removing the medium from the cells, we washed once with phosphate-buffered saline (PBS), followed by incubating them for 48 h with different pharmaceutics diluted in cell culture medium, including Se NPs, Pt NPs, and Pt–Se NPs with different concentrations. Then, CCK-8 was employed to evaluate the cell viability by using microplate to measure the absorbance at 450 nm. Meanwhile, calcein-AM/PI assay was used to analyze the cell live/dead staining by a fluorescence microscope, excited by a 490-nm laser, observed at wavelengths of 535 nm, and displayed in blue. Cell viability was calculated by ImageJ.

### Cellular uptake assay

The cellular uptake of Pt–Se NPs was assessed using a confocal laser scanning fluorescence microscope (Leica, Heidelberg, Germany). Chondrocytes in the exponential growth phase were cultivated on chambered coverslips. Following overnight incubation, the cells were exposed to free Cy5.5 and DSPE-Cy5.5 encapsulated Pt–Se NPs and subsequently incubated for 2 or 4 h. Subsequently, PBS was used to wash the cells three times, followed by fixation with 4% paraformaldehyde (PFA) for 20 min. To stain the cells, Hoechst 33342 was applied to label cell nuclei for 10 min. Images were acquired using different channels and merged into composite pictures.

### Collection of macrophage CM

Activated macrophages were further investigated by using a coculture system. M1-type macrophages were induced by RAW264.7 cells exposed to 10 μg/ml LPS for 12 h and then treated as described above. The obtained macrophage conditioned medium (CM) was at centrifugated at 1000 g for 5 minutes and storage at −80°C. Further studies were performed by dilution of macrophage CM 1:1 in serum-free medium, coculturing it with chondrocytes.

### Intracellular ROS detection

The intracellular ROS-scavenging effect of Se, Pt, and Pt–Se NPs was evaluated by flow cytometry and fluorescence microscopy. RAW264.7 cells at a density of 2 × 10^5^ cells/well were seeded in a six-well plate and treated as the following groups: (a) M1: RAW264.7 cells treated by stimulation with H_2_O_2_ (200 μM) for 12 h; (b) RAW: normal RAW264.7 cells without any treatment; (c) M1 + Pt NPs: RAW264.7 cells induced by H_2_O_2_ for 12 h followed by Pt NP addition; (d) M1 + Se NPs: RAW264.7 cells interacted with H_2_O_2_ for 12 h followed by Se NP addition; (e) M1 + Pt–Se NPs: RAW264.7 cells interacted with H_2_O_2_ for 12 h followed by Pt–Se NP addition. Chondrocytes with a density of 2 × 10^5^ were also set as five groups: RAW-CM, M1-CM, M1 + Pt-CM, M1 + Se-CM, and M1 + Pt–Se-CM. Then, all the groups were incubated for 24 h, washed with PBS, and stained for 30 min with 10 μM DCFH-DA probe. After that, the samples were collected. We acquired the fluorescent images using a fluorescence microscope. To quantify the ROS level in RAW264.7 cells, the fluorescence intensity was measured by flow cytometry (FACS Verse, BD, USA).

### Detection of intracellular NO levels

NO was detected intracellularly using a Nitric Oxide Synthase Assay Kit. In brief, the macrophage culture medium was removed, and 1 ml of a DAF-FM DA probe was added to each well to a final concentration of 5 μM. Cells were further incubated at 37 °C for 20 min and washed with PBS to remove DAF-FM DA that had not entered the cells. Then, the cells were examined under a fluorescence microscope, and fluorescence intensity analysis was performed using ImageJ to determine the average intensity.

### Immunofluorescent staining

After seeding into six-well plates, the cells were treated as described above. After washing three times with PBS, cells were fixed for 15 min in 4% PFA. Subsequently, the cells were treated by BSA (1%) for 30 min. After overnight incubation at 4 °C with primary antibody (INOS, CD206, MMP-13), secondary antibody [donkey anti-goat immunoglobulin G (IgG)-FITC] was added for 1 h at room temperature. Subsequently, these cells were stained with 4′,6-diamidino-2-phenylindole (DAPI) (1 μg/ml) for 5 min after washing with PBS. Finally, an antifade mounting medium was used to seal all samples, and then fluorescence images were acquired with a fluorescence microscope and quantified with ImageJ.

### qRT-PCR analysis

In order to quantify RT-PCR results, RAW264.7 cells were plated onto six-well plates and activated for 12 h with LPS (10 μg/ml). Then, 100 μg/ml of Se NPs, Pt NPs, and Pt–Se NPs was added. After culturing for 24 h, cells were collected and total RNA was extracted by a Total RNA Extraction Kit. cDNA was synthesized with Fermentas Company reverse transcription kit. qRT-PCR was used to detect mRNA expression levels for IL-1β, IL-6, iNOS, TNF-α, CD206, and IL-10. The primers used were illustrated in Table S2. Chondrocytes treated with the CM were then determined the levels of mRNAs (including IL-6, MMP-3, MMP-13, iNOS, TNF-α, ACAN, and Col2A1) by the same method as described above. The primers used were illustrated in Table S2. Gene expression levels were normalized to GAPDH mRNA expression levels and calculated using the 2^−ΔΔCt^ method.

### Intracellular calcium ion detection

A Calcium Colorimetric Assay Kit (Fluo-3 AM) was used to tested intracellular calcium ion. The chondrocytes plated in 12-well plates were cultured with different groups of CM for 24 h. Then, 100 μl of 3 μM Fluo-3 AM working solution was added to each well. Using a microplate reader, we measured the fluorescence signal intensity with a microplate reader under 520 nm after incubation at 37 °C for 30 min.

### Intracellular mitochondrial activity test

After chondrocytes were treated with different groups of CM as described above, cells were incubated with JC-1 solution at 37 °C for 20 min after washing with PBS. Then, the supernatant was removed and cells were washed with JC-1 staining buffer. After that, fluorescence images were taken using a fluorescence microscope.

### In vivo therapeutic effects

#### IVIS imaging of NPs

The OA model was successfully established 4 weeks after ACLT. In order to assess the persistence of NPs within the articular cavity, an IVIS imaging system was employed. Pt–Se NPs, which were coated with DSPE-NH_2_ and labeled with red fluorescence Cy5.5, were introduced into the joint cavity of OA rats to assess the intensity of the fluorescent signal. Free Cy5.5 and Pt–Se/DSPE-Cy5.5 were injected into the joint cavity of Sprague-Dawley rats at 0 d (within 30 min after injection), 1 d, 3 d, 5 d, 7 d, and 14 d, respectively, and fluorescence intensity was detected by IVIS imaging system (Aniview100, China). The fluorescence intensity was analyzed using the imaging system.

#### Immunofluorescence staining of tissues

In order to assess the polarization conversion of M1 and M2 in the synovial tissue after treatment with various types of NPs, we opted to utilize iNOS as an indicator for M1 macrophages and CD206 as a marker for M2 macrophages. We subjected the tissues to an overnight incubation with anti-iNOS and anti-CD206 antibodies (1:200), followed by staining with fluorescent secondary antibodies and DAPI to visualize the samples under a confocal laser scanning fluorescence microscope.

#### Histological observation

Sixty male Sprague-Dawley rats (150 ± 180 g) were used for the in vivo experiment, with the experimental animal ethics provisions approved by the Animal Ethics Committee of Guangxi Medical University (No. 202108014). Sprague-Dawley rats were conducted with ACLT for 4 weeks to establish OA animal model. In the sham group, only skin and joint capsule were cut. The rats were randomly set as five groups: sham: no further treatment, OA: articular injected with 200 μl of PBS buffer; OA + Pt: articular injected with 200 μl of Pt NPs (equivalent concentration of Pt in Pt–Se); OA + Se: articular injected with 200 μl of Se NPs (equivalent concentration of Se in Pt–Se); OA + Pt–Se: articular injected with 200 μl of Pt–Se NPs (100 μg/ml). The rats were injected twice a week with the above solution and sacrificed with an overdose of sodium pentobarbital at 4 and 8 weeks after treatment, respectively. The knee joints were harvested, photographed, and scored in accordance with Pelletier’s scoring [60]. Then, the joints of all the groups were fixed with 4% PFA for 48 h and then decalcified in EDTA by ultrasonic decalcifying unit for 3 weeks. Tissue samples were stained with H&E and S&F after sectioning. Immunofluorescence staining was used to observe the polarization of synovial macrophages in vivo. Then, tissue sections were viewed through a light microscope (BX53, Olympus, Japan) and laser scanning confocal microscope (STELLARIS5, Leica, Germany) and assessed according to the Osteoarthritis Research International Association (OARSI) scores.

### Statistical analysis

There were at least three replicate measurements in this study, and each set was presented as mean ± SD. A statistical significance test was performed using SPSS software’s one-way analysis of variance (ANOVA), and pairwise comparison between groups was performed using least-significant difference (LSD) *t* test.

## Data Availability

All data needed to evaluate the conclusions in the paper are present in the paper and/or the Supplementary Materials. Additional data related to this paper may be requested from the authors.
