# Erratum to “Pt–Se Hybrid Nanozymes with Potent Catalytic Activities to Scavenge ROS/RONS and Regulate Macrophage Polarization for Osteoarthritis Therapy”

**DOI:** 10.34133/research.0395

**Published:** 2024-06-21

**Authors:** Hong Wei, Hongjun Huang, Haoqiang He, Yuanming Xiao, Lu Chun, Zhiqiang Jin, Hanyang Li, Li Zheng, Jinmin Zhao, Zainen Qin

**Affiliations:** ^1^ Guangxi Engineering Center in Biomedical Materials for Tissue and Organ Regeneration & Collaborative Innovation Center of Regenerative Medicine and MedicalBioResource Development and Application Co-constructed by the Province and Ministry, The First Affiliated Hospital of Guangxi Medical University, Nanning 530021, China.; ^2^ Department of Orthopaedics, Affiliated Hospital of Guilin Medical University, Guilin 541000, China.; ^3^ Life Sciences Institute, Guangxi Medical University, Nanning 530021, China.; ^4^ Department of Orthopaedics Trauma and Hand Surgery, The First Affiliated Hospital of Guangxi Medical University, Nanning 530021, China.; ^5^ School of Materials and Environment, Guangxi Minzu University, Nanning, Guangxi 53000, China.; ^6^ Guangxi Key Laboratory of Regenerative Medicine, The First Affiliated Hospital of Guangxi Medical University, Nanning 530021, China.

In the Research Article “Pt–Se hybrid nanozymes with potent catalytic activities to scavenge ROS/RONS and regulate macrophage polarization for osteoarthritis therapy” [1], the authors made an inadvertent error where 2 images was mistakenly included in Fig. 9F and Fig. S5A. Specifically, during the figure assembly process, the images of “CD206” in 4W + osteoarthritis (OA) + PtSe nanoparticle (NP) group were misused as images of “CD206” in 8W + OA + Se NP group in Fig. 9F. Moreover, in Fig. S5A, another image of “0 d” in the “free Cy5.5” group was inadvertently chosen as the “0 d” image in the “PtSe/DSPE NP” (PtSe coated with DSPE-NH_2_) group, so they appear to be remarkably similar. The authors want to assure readers that this issue has been promptly addressed, and the corrected image of the Fig. 9F and Fig. S5A is below. Importantly, it should be noted that this error does not affect the scientific conclusions drawn in the study. The authors sincerely apologize for any inconvenience caused by this oversight.

**Fig. 9.**
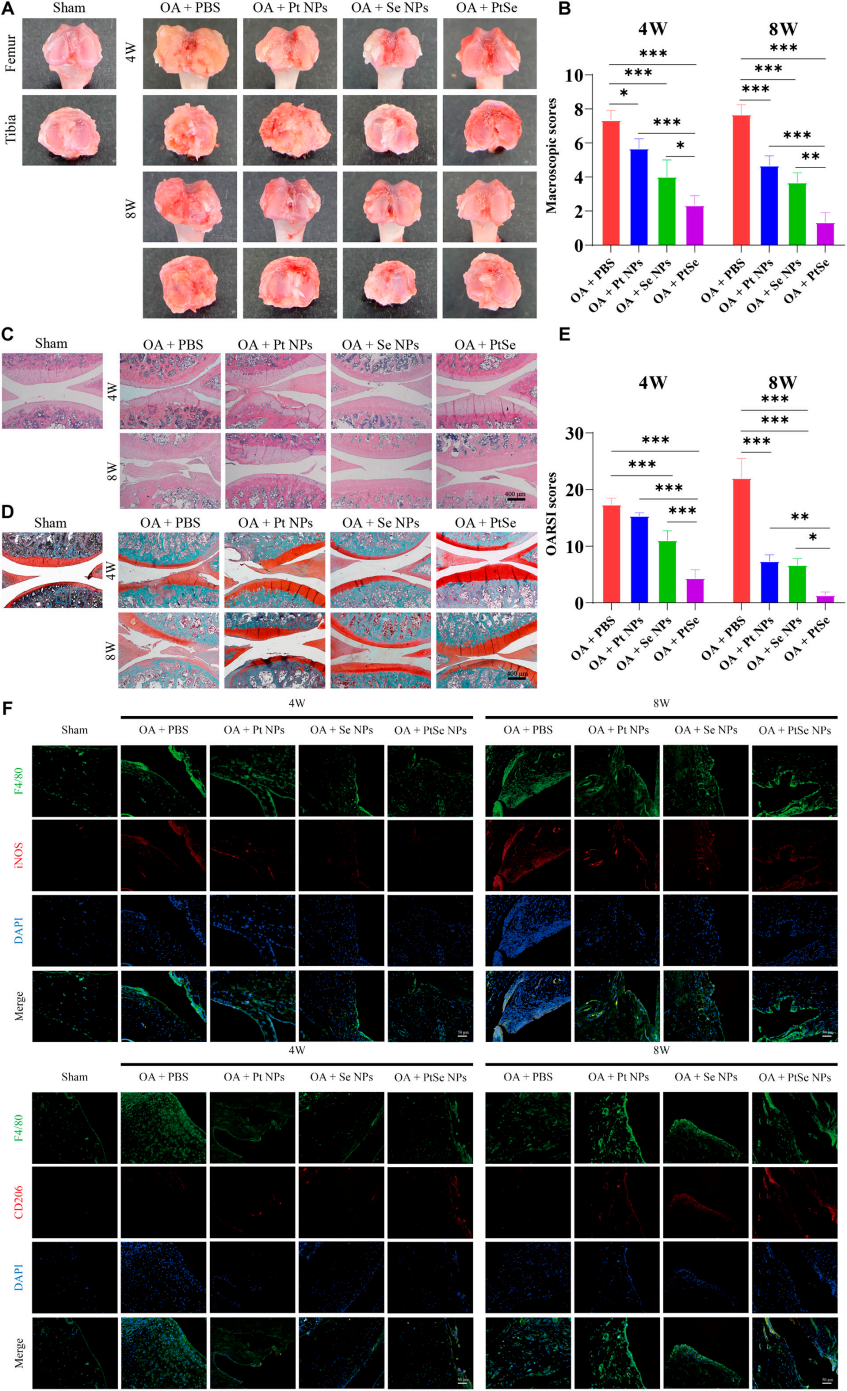
Effects of NPs on OA in vivo. Macroscopic observation (A) and corresponding macroscopic scores (B) of OA articular cartilage after 4 and 8 weeks of treatment with the intra-articular injection of NPs. Hematoxylin and eosin staining (C), Safranin O-Fast Green staining (S&F) (D), and corresponding histological scores (E) of articular cartilages after treatment with NPs. (F) The polarization of macrophages in joint synovial membrane was observed by immunofluorescence staining. Macrophages were labeled with F4/80 (green), and M1-type macrophage-related markers were detected by inducible nitric oxide synthase (iNOS) (red), while M2-type macrophage-related markers were detected by CD206 (red). PBS, phosphate-buffered saline; DAPI, 4 DAPI, sphate-buffered salin. Original magnification, ×100. Scale bars, 400 μm. *n* = 3, *P < 0.05, **P < 0.01, and ***P < 0.001.

## Supplementary Materials

Supplementary 1 Fig. S5. IVIS (Small Animal Imaging System) imaging was performed to detect the retention time of NPs in vivo.
