# Supplementary material for: Pt–Se Hybrid Nanozymes with Potent Catalytic Activities to Scavenge ROS/RONS and Regulate Macrophage Polarization for Osteoarthritis Therapy
Source: Research (Wash D C). 2024 Feb 26;7:0310. doi: 10.34133/research.0310 (PMC10895487; doi:10.34133/research.0310)
Supplement: Supplementary 1 — Figs. S1 to S5 Tables S1 and S2 [file research.0310.f1.zip › Revised supporting information20231226.docx]

**Supplementary information**

**Pt-Se Hybrid Nanozymes with Potent Catalytic Activities to Scavenge ROS/NO and Regulate Macrophage Polarization For Osteoarthritis Therapy**

Hong Wei^a^, Hongjun Huang^a,b^, Yuanming Xiao^a,c,d^, Lu Chun^e^, Zhiqiang Jin^a.d^, Hanyang Li^b^, Haoqiang He^a^, Li Zheng^a^*, Jinmin Zhao^a,d,f^* Zainen Qin^a,f*^

^a^Guangxi Engineering Center in Biomedical Materials for Tissue and Organ Regeneration, Collaborative Innovation Centre of Regenerative Medicine and Medical BioResource Development and Application, The First Affiliated Hospital of Guangxi Medical University, Nanning, 530021, China

^b^Department of Orthopaedics, Affiliated Hospital of Guilin Medical University, Guilin, 541000, China

^c^Life Sciences Institute, Guangxi Medical University, Nanning, 530021, China

^d^Department of Orthopaedics Trauma and Hand Surgery, The First Affiliated Hospital of Guangxi Medical University, Nanning, 530021, China

^e^School of Materials and Environment, Guangxi Minzu University, Nanning, Guangxi, 53000, PR China

^f^Guangxi Key Liboratory of Regenerative Medicine, The First Affiliated Hospital of Guangxi Medical University, Nanning, 530021, China

Hong Wei and Hongjun Huang contributed equally to this work.

* Co-Corresponding authors: Zainen Qin, Li Zheng, Jinmin Zhao

*To whom correspondence should be addressed: qinzainen@sr.gxmu.edu.cn, [zhengli224@163.com](mailto:zhengli224@163.com), [zhaojinmin@126.com](mailto:zhaojinmin@126.com),

Guangxi Engineering Center in Biomedical Materials for Tissue and Organ Regeneration, Nanning, 530021, China, Fax: +86-0771-5540585


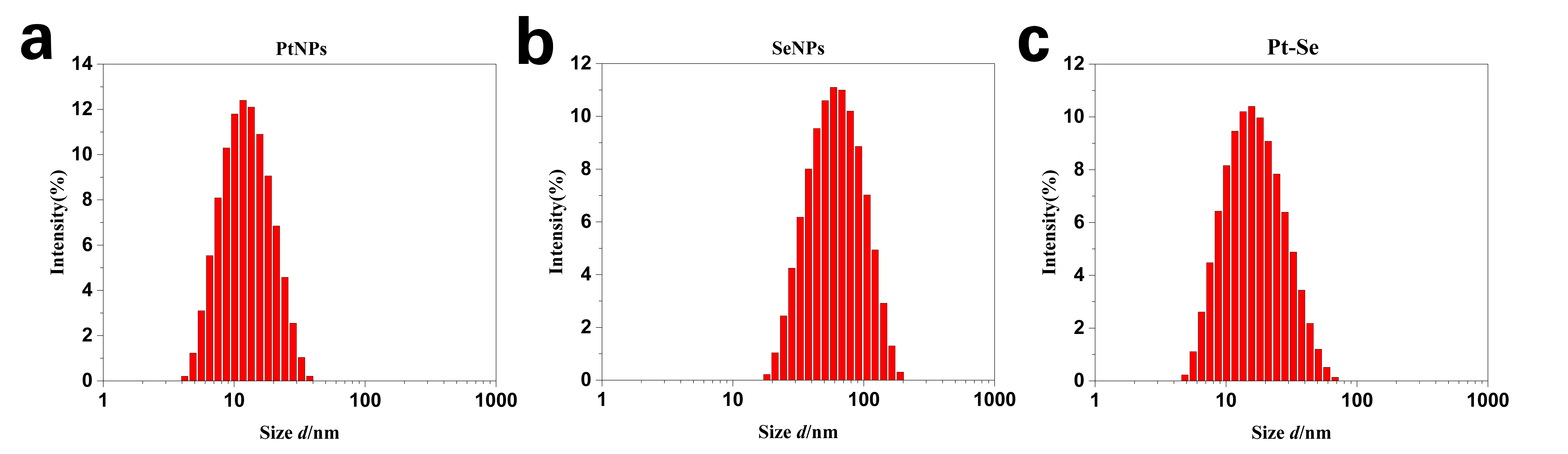


**Figure S1**. DLS image of Pt NPs (a), Se NPs (b) and Pt-Se nanoparticles (c).


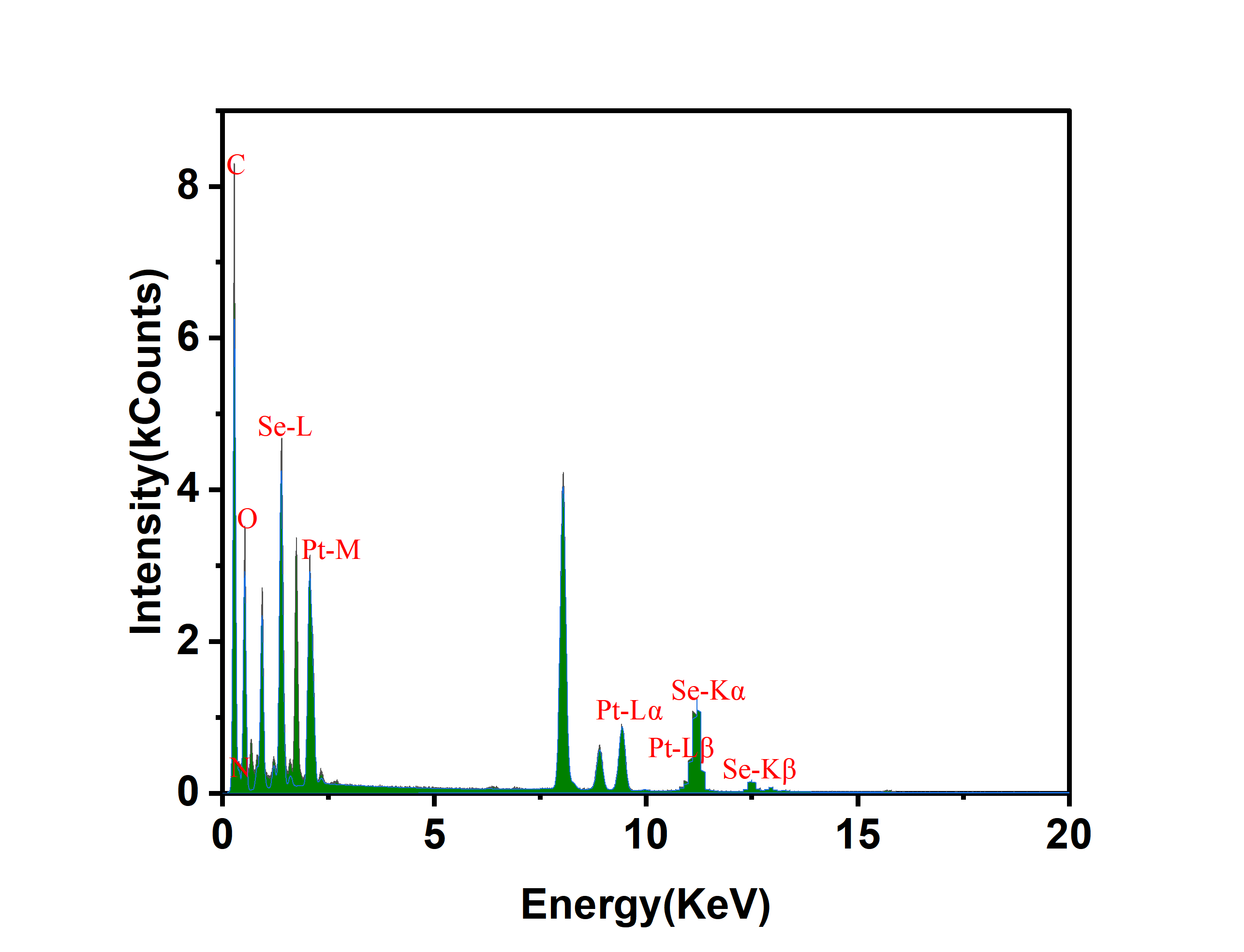


**Figure S2**. EDS element distribution diagram of Pt-Se nanoparticles.





**Figure S3**. (a) The viability of chondrocytes was detected by a CCK-8 assay after treatment with NPs for 24 h. (b) The expression of genes related to inflammation in chondrocytes was detected by qRT-PCR after NPs treatment.


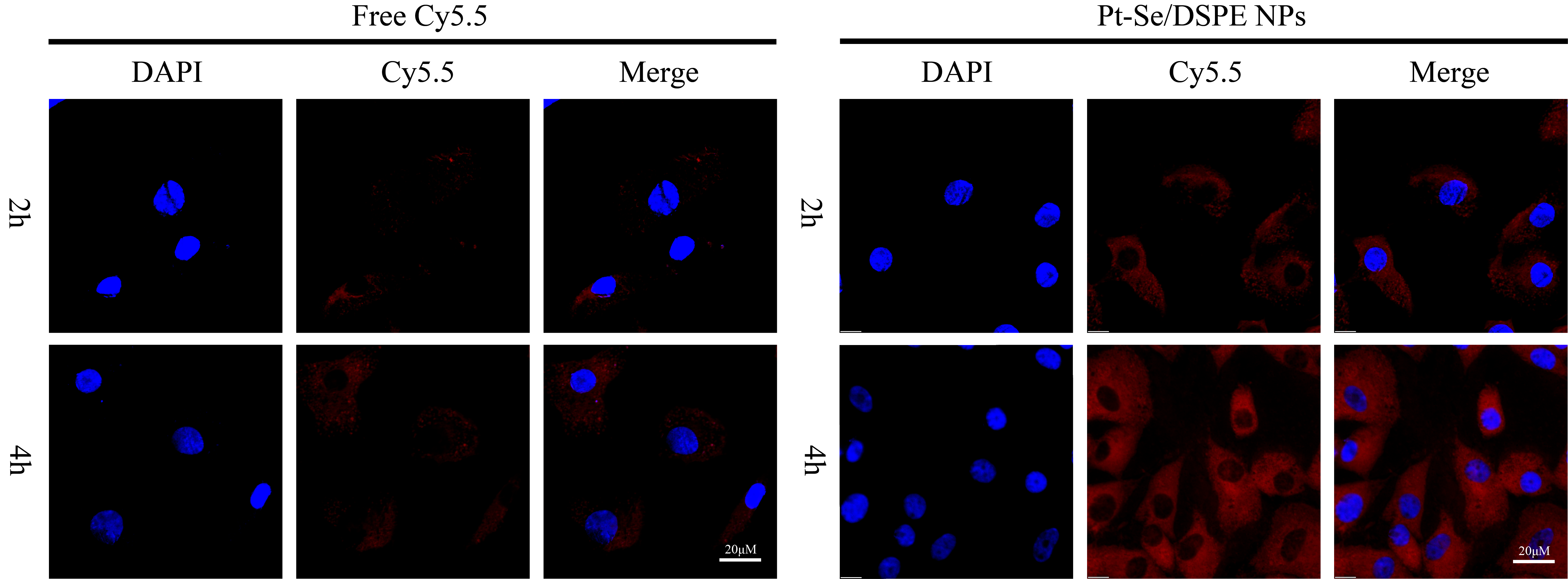


**Figure S4**. Fluorescence images of chondrocytes uptake of Pt-Se nanoparticles. Pt-Se/DSPE NPs were labeled with Cy5.5 (red) and the cell nucleus were labeled with DAPI (blue), and fluorescence intensity of cells was observed at 2 h and 4 h, respectively. (Original magnification: 200x, Scale bar: 20μm)


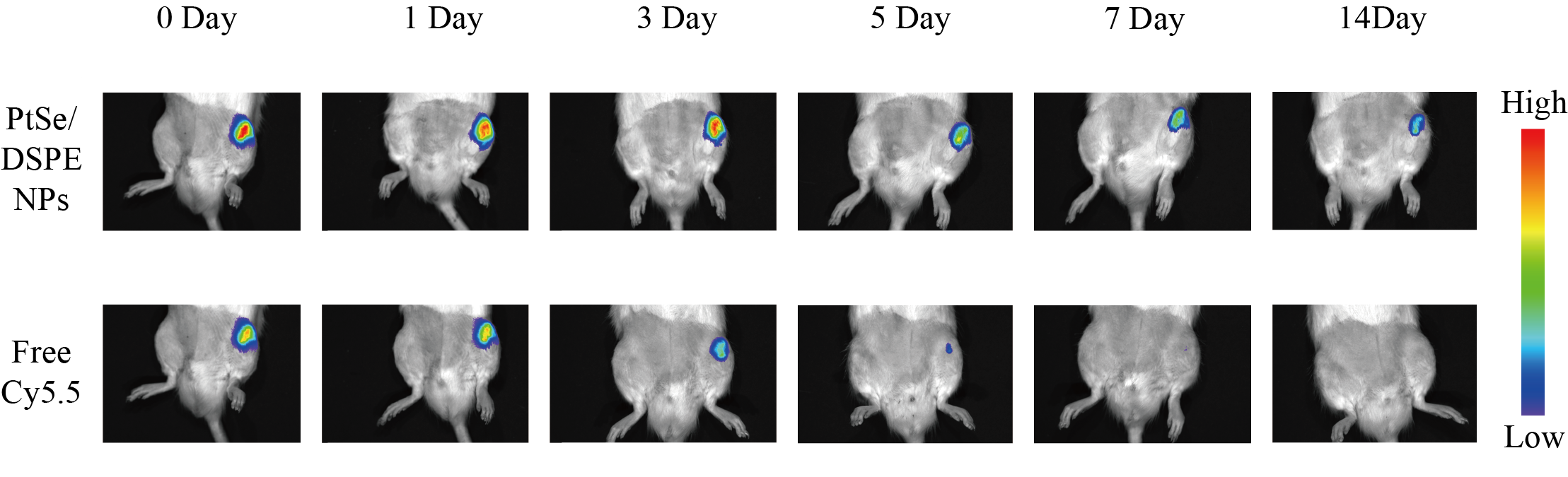


**Figure S5**. IVIS imaging was performed to detect the retention time of nanoparticles in *vivo*. a. After injection of Cy5.5 labeled Pt-Se/DSPE NPs into the articular cavity, fluorescence imaging in vivo was obtained in OA rats at 0, 1, 3, 5, 7, and 14 days. b. Quantitative analysis for fluorescence of Pt-Se NPs in the joints after IA-injection for 0, 1, 3, 5, 7, and 14 days (relative fold-changes to first time point were calculated and shown as mean±SD, n=3).

**Table S1.** The ratio of carbon, nitrogen, oxygen, platinum, and selenium of Pt-Se NPs by EDS.

| Name | C% (±SD) | N% (±SD) | O% (±SD) | Pt% (±SD) | Se% (±SD) |
| --- | --- | --- | --- | --- | --- |
| Pt-Se | 69.06±6.20 | 1.88±0.43 | 17.76±3.86 | 7.64±1.18 | 3.64±0.50 |

**Table S2**. Gene primers sequence used in the article.

| Gene | Forward sequence (5’ to 3’) | Reverse sequence (3’ to 5') |
| --- | --- | --- |
| GAPDH | ACTTGAAGGGTGGAGCCAAA | GCCCTTCCACAATGCCAAAG |
| Arg-1 | CATATCTGCCAAGGACATCG | GGTCTCTTCCATCACTTTGC |
| CD206 | AGGGTGCGGTACACTAACTG | TCTGACTCTGGACACTTGCC |
| IL-10 | GAGAAGCATGGCCCAGAAATC | GAGAAATCGATGACAGCGCC |
| iNOS | GTTCTCAGCCCAACAATACAAGA | GTGGACGGGTCGATGTCAC |
| IL-1β | TGCCACCTTTTGACAGTGATG | ATGTGCTGCTGCGAGATTTG |
| IL-6 | GAGAAATCGATGACAGCGCC | GATGAATTGGATGGTCTTGGTCC |
| TNF-α | GGAGGGAGAACAGCAACTCC | TCTGCCAGTTCCACATCTCG |
| IL-1β | GCACAGTTCCCCAACTGGTA | GGAGACTGCCCATTCTCGAC |
| IL-6 | ACAAGTCCGGAGGAGGAGACT | ACAGTGCATCATCGCTGTTC |
| TNF-α | GGAGGGAGAACAGCAACTCC | TCTGCCAGTTCCACATCTCG |
| iNOS | GGTGAGGGGACTGGACTTTTAG | TCTCCGTGGGGCTTGTAGTT |
| MMP-3 | GGCTGTGTGCTCATCCTACC | TGGAAAGGTACTGAAGCCACC |
| MMP-13 | GGACAAAGACTATCCCCGCC | GGCATGACTCTCACAATGCG |
| ACAN | GACAAGGACGAGTTCCCTGG | CTCCGGGGATGTGGCATAAA |
| Col2A1 | ATTGCCTACCTGGACGAAGC | GACAGGCCCTATGTCCACAC |
