# Supplementary figures and images for: Pt–Se Hybrid Nanozymes with Potent Catalytic Activities to Scavenge ROS/RONS and Regulate Macrophage Polarization for Osteoarthritis Therapy
Source: Research (Wash D C). 2024 Feb 26;7:0310. doi: 10.34133/research.0310 (PMC10895487; doi:10.34133/research.0310)

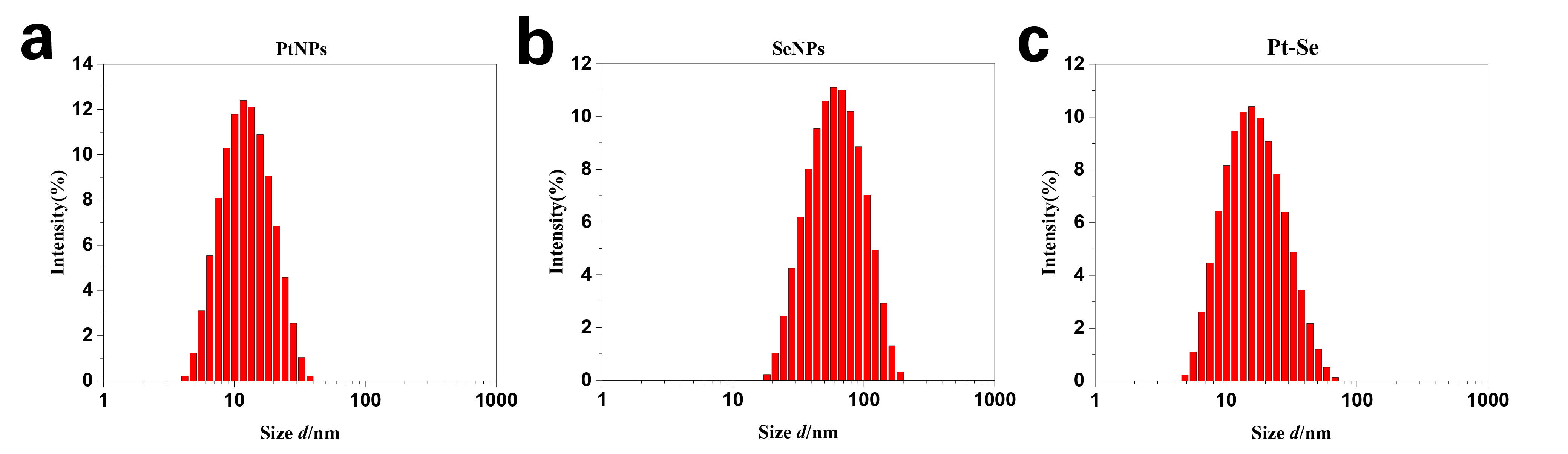

Supplement: Supplementary 1 — Figs. S1 to S5 Tables S1 and S2 [file research.0310.f1.zip › Figure S1.tif]

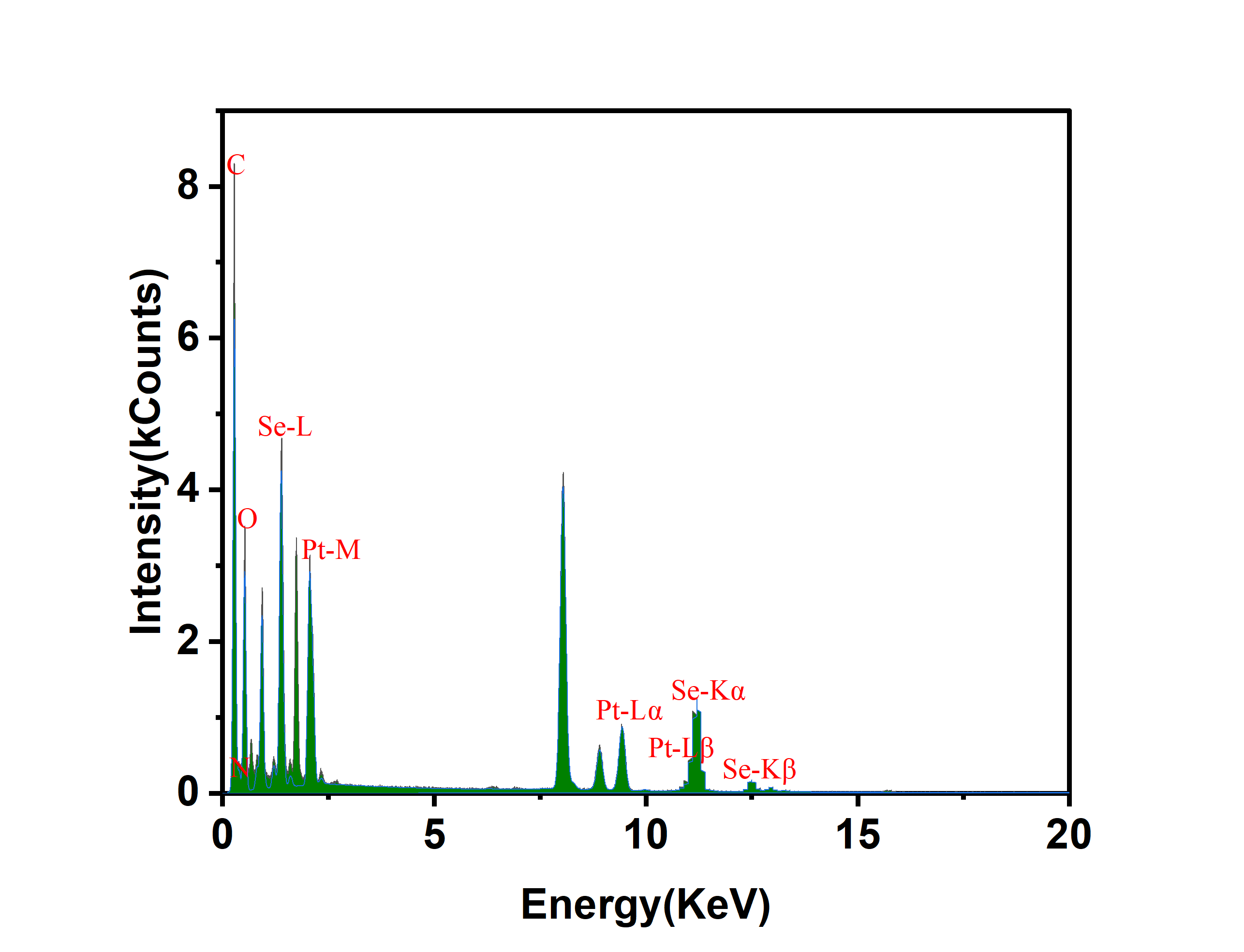

Supplement: Supplementary 1 — Figs. S1 to S5 Tables S1 and S2 [file research.0310.f1.zip › Figure S2.png]

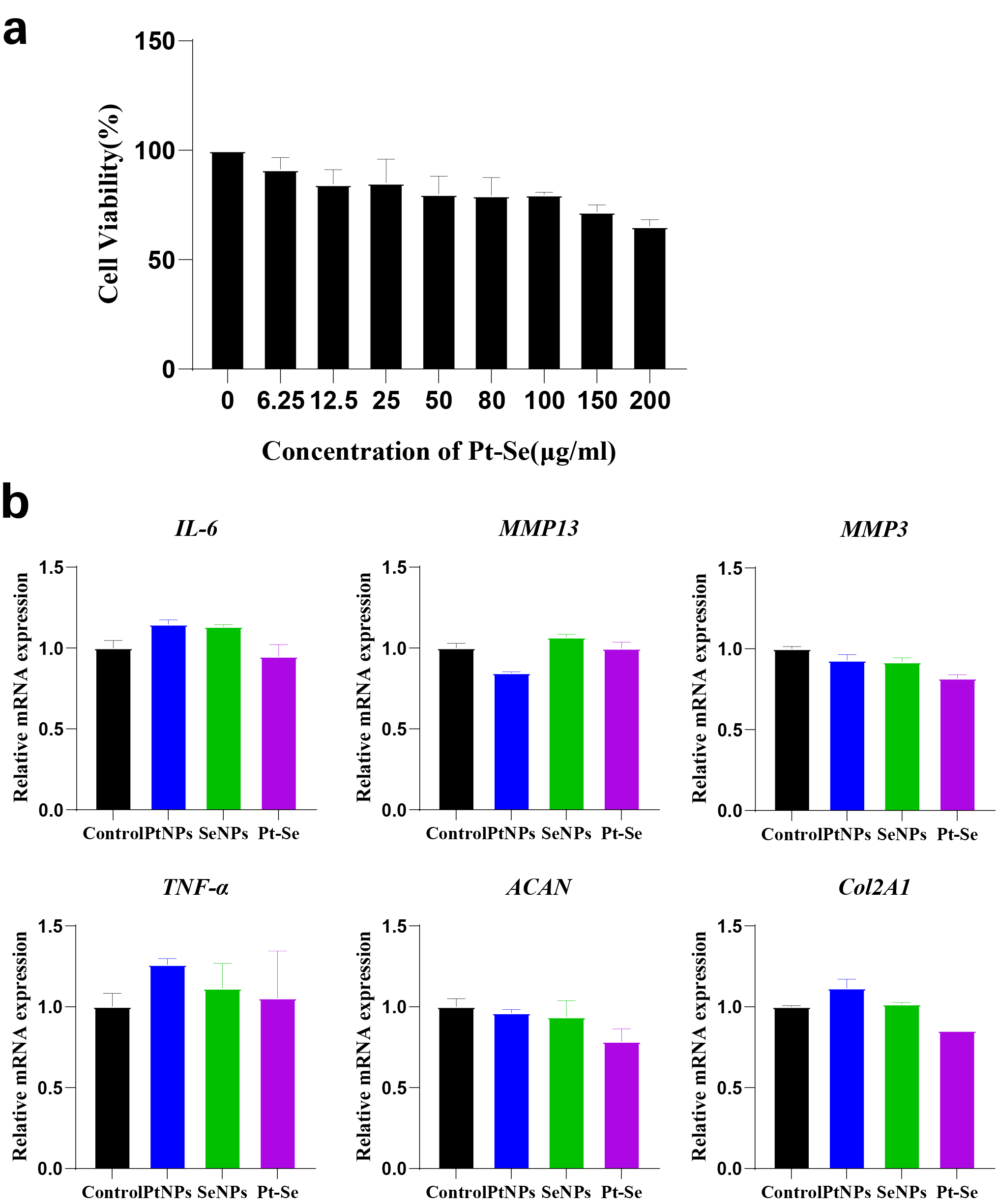

Supplement: Supplementary 1 — Figs. S1 to S5 Tables S1 and S2 [file research.0310.f1.zip › Figure S3.tif]

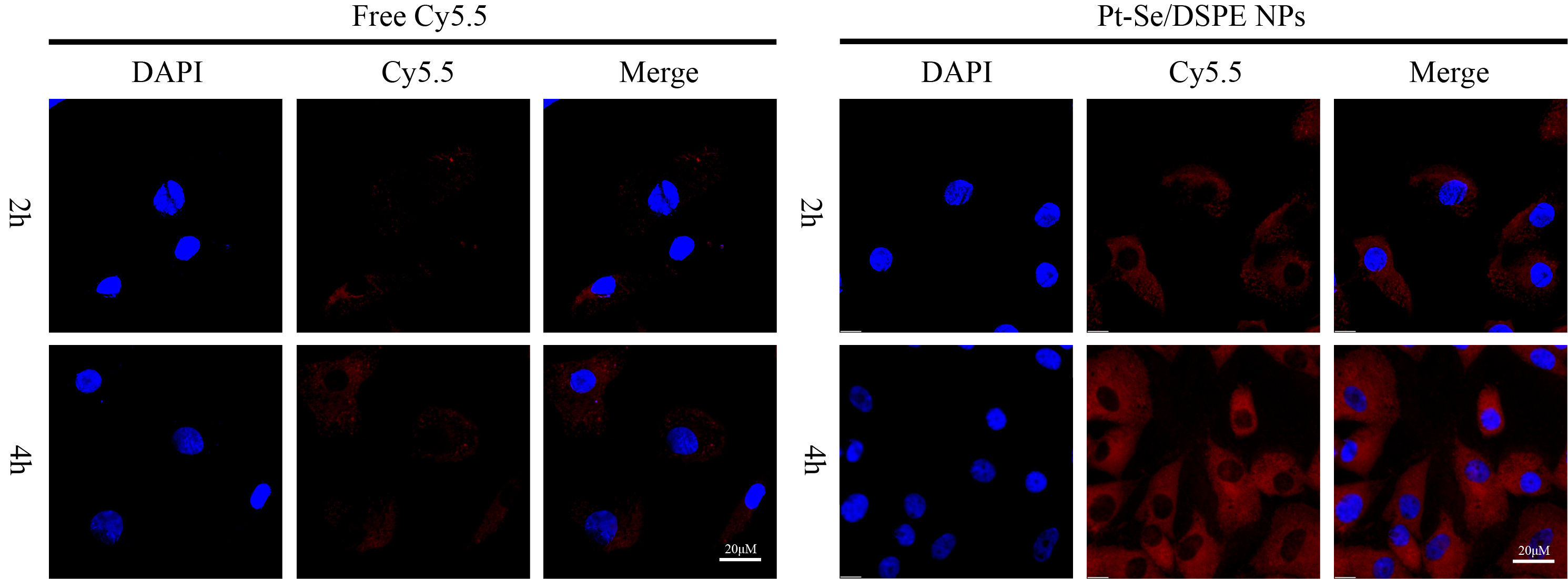

Supplement: Supplementary 1 — Figs. S1 to S5 Tables S1 and S2 [file research.0310.f1.zip › Figure S4.tif]

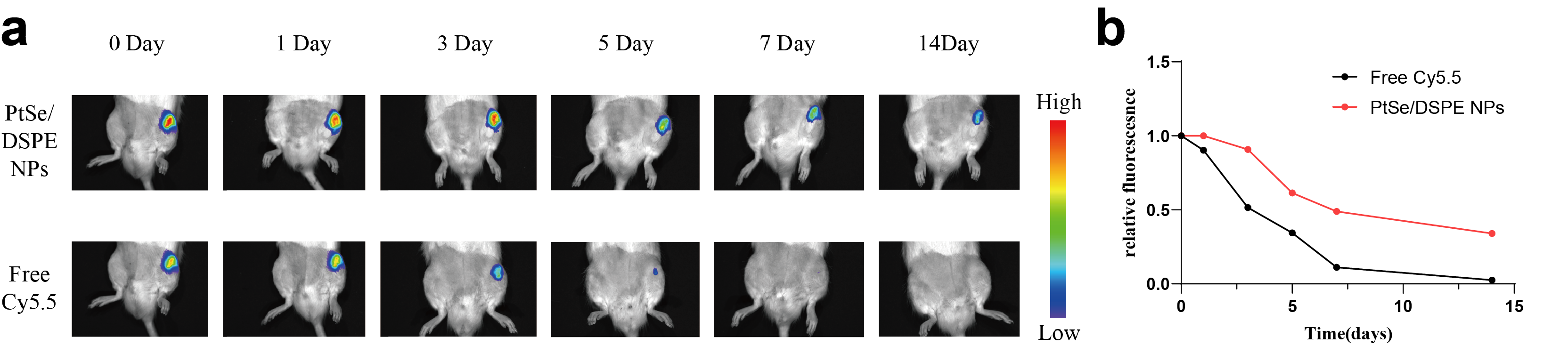

Supplement: Supplementary 1 — Figs. S1 to S5 Tables S1 and S2 [file research.0310.f1.zip › Figure S5.tif]
