# Supplementary material for: Erratum to “Pt–Se Hybrid Nanozymes with Potent Catalytic Activities to Scavenge ROS/RONS and Regulate Macrophage Polarization for Osteoarthritis Therapy”
Source: Research (Wash D C). 2024 Jun 21;7:0395. doi: 10.34133/research.0395 (PMC11419331; doi:10.34133/research.0395)
Supplement: Supplementary 1 — Fig. S5. IVIS (Small Animal Imaging System) imaging was performed to detect the retention time of NPs in vivo. [file research.0395.f1.zip › Hong Wei-Erratum - SM.docx]

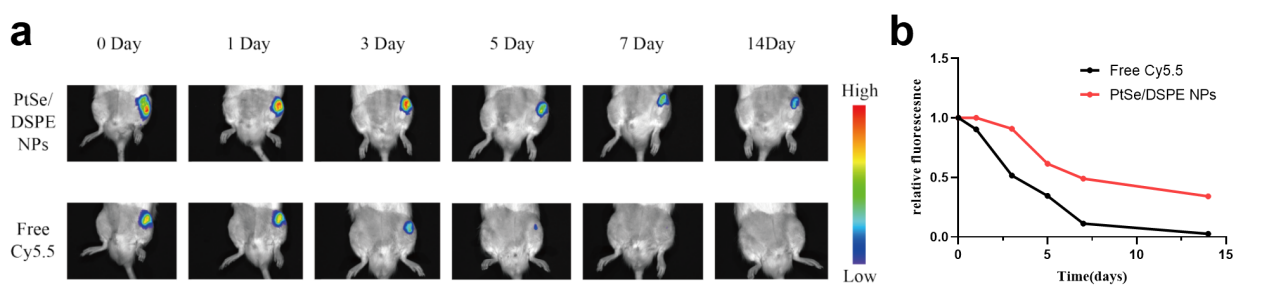


Figure S5. IVIS imaging was performed to detect the retention time of nanoparticles in vivo. a. After injection of Cy5.5 labeled Pt-Se NPs into the articular cavity, fluorescence imaging in vivo was obtained in OA rats at 0, 1, 3, 5, 7, and 14 days. b. Quantitative analysis for fluorescence of Pt-Se NPs in the joints after IA-injection for 0, 1, 3, 5, 7, and 14 days (relative fold-changes to first time point were calculated and shown as mean±SD, n=3).
