# Supplementary figures and images for: Erratum to “Pt–Se Hybrid Nanozymes with Potent Catalytic Activities to Scavenge ROS/RONS and Regulate Macrophage Polarization for Osteoarthritis Therapy”
Source: Research (Wash D C). 2024 Jun 21;7:0395. doi: 10.34133/research.0395 (PMC11419331; doi:10.34133/research.0395)

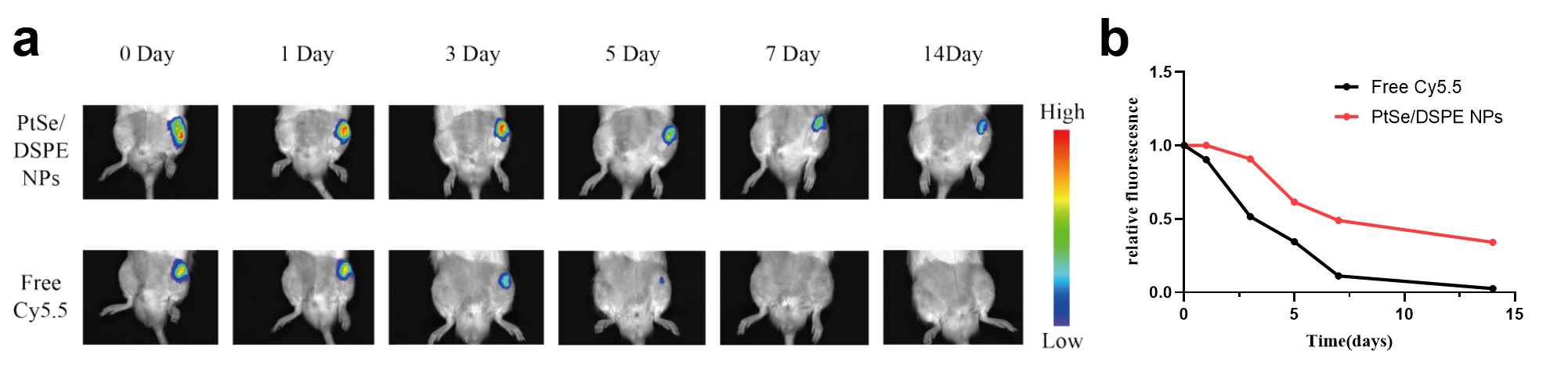

Supplement: Supplementary 1 — Fig. S5. IVIS (Small Animal Imaging System) imaging was performed to detect the retention time of NPs in vivo. [file research.0395.f1.zip › Figure S5.png]
